# Lactate-mediated macrophage polarization promotes splenomegaly in acute erythroleukemia

**DOI:** 10.1038/s41419-026-08612-5

**Published:** 2026-03-25

**Authors:** Mingyue Yang, Dan Xie, Yanlong Zhang, Yi Ye, Suwen Yang, Hongqian Zhu, Sha Cheng, Jia Yu, Ningning Zan, Shengwen Huang, Heng Luo

**Affiliations:** 1 Guizhou University Medical College, Guiyang, China; 2 State Key Laboratory of Discovery and Utilization of Functional Components in Traditional Chinese Medicine, Guizhou Medical University, Guiyang, China; 3 Department of Medical Genetics, Guizhou Provincial People’s Hospital, Guiyang, China; 4 Natural Products Research Center of Guizhou Province, Guiyang, China; 5 Department of Haematology, Guizhou Provincial People’s Hospital, Guiyang, China

**Keywords:** Cell biology

## Abstract

Acute erythroleukemia (AEL) is a rare and highly aggressive subtype of acute myeloid leukemia (AML) that is often accompanied by splenomegaly in some patients. Using the Friend murine leukemia virus clone 57 (F-MuLV clone 57) mouse model, we observed lactate accumulation in the spleens of mice with late-stage disease. Proteomic profiling indicated dysregulation of the glycolysis/gluconeogenesis pathway and aberrant activity of its key enzymes. In vitro, lactate alone directly induced macrophage polarization to the pro-inflammatory M1 phenotype. This lactate-rich milieu reprograms macrophage function, favoring M1 polarization. A self-reinforcing cycle thus emerges in the AEL splenic microenvironment: lactate drives M1 polarization, and these M1 macrophages subsequently elevate their glycolytic activity, amplifying local lactate production that further promotes M1 polarization. In vivo, pharmacological inhibition of lactate production with Oxamate disrupted this cycle, reversed pathogenic M1 polarization, ameliorated splenomegaly, and extended survival. These results identify lactate as a key immunomodulatory factor in the splenic microenvironment that accelerates AEL progression. Targeting this lactate-driven metabolic-immune axis represents a novel adjunctive strategy for mitigating splenomegaly and disease progression in AEL.

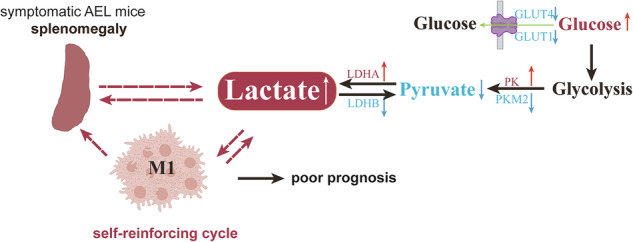

## Introduction

Acute erythroleukemia (AEL), a rare and aggressive subtype of acute myeloid leukemia (AML), is characterized by the predominant proliferation of erythroid precursors and carries an extremely poor prognosis, with a median survival typically under six months [1–4]. A prominent clinical feature of AEL is splenomegaly, a common manifestation in leukemia. This condition arises from the abnormal infiltration of leukemic cells into the spleen. Splenomegaly can precipitate severe complications and is associated with increased mortality [5–9]. However, the mechanisms driving leukemia-associated splenomegaly and its optimal treatment strategies are not fully elucidated [10].

The Friend Murine Leukemia Virus (F-MuLV)-induced murine erythroleukemia model serves as a critical system for studying the multistage pathogenesis of erythroid malignancies. This model faithfully reproduces major clinical hallmarks of human AEL, including blocked erythroid differentiation, splenomegaly, and anemia [11–14]. Following F-MuLV infection, susceptible neonatal mouse strains such as Balb/c and NIH Swiss develop a biphasic disease: an initial preleukemic phase of non-malignant compensatory erythroid hyperplasia, followed by a later phase of malignant transformation characterized by leukemic cells and defective erythropoiesis. Both phases present shared clinical features, particularly splenomegaly and anemia [13, 15, 16]. Existing studies, however, have largely concentrated on global genetic changes and dysregulated signaling pathways, such as Fli-1 activation and TP53 inactivation [17, 18]. The mechanisms that specifically drive splenomegaly and the accompanying metabolic alterations during disease progression remain poorly understood.

The Warburg effect represents a hallmark of cancer. Tumor cells preferentially metabolize glucose through anaerobic glycolysis, generating energy via glycolysis and lactate fermentation even under aerobic conditions [19, 20]. In AML, elevated glycolytic flux meets the demands of highly proliferative blasts and is linked to poor prognosis and ex vivo chemoresistance [21–23]. Lactate, the end product of glycolysis, is now understood not as a mere waste product but as a key oncometabolite. Emerging evidence identifies lactate as a critical nutrient within the tumor niche [24]. Functioning as a signaling molecule, lactate can remodel the tumor microenvironment, influence immune cell function, and drive epigenetic reprogramming through histone lactylation [25, 26]. Moreover, lactate utilization aids leukemia cells in maintaining metabolic homeostasis and evading therapy, whereas inhibiting lactate metabolism effectively suppresses their proliferation [27, 28], highlighting the therapeutic potential of targeting this axis. However, such metabolic reprogramming has seldom been reported in AEL, and its functional role in leukemia-associated organomegaly remains entirely unexplored.

In this study, we employed an F-MuLV-induced AEL mouse model to investigate the role of metabolic reprogramming in disease progression. Splenic tissues from symptomatic mice showed pronounced lactate accumulation, and proteomic analysis of the spleen revealed significant dysregulation of the glycolysis/gluconeogenesis axis. Inhibiting lactate production in vivo substantially alleviated splenomegaly and extended survival. Mechanistic studies revealed that the mTOR and STAT1 signaling pathways regulate lactate production and M1-like macrophage polarization. These results demonstrate that lactate accumulation correlates with disease severity and that targeting lactate homeostasis ameliorates key disease phenotypes, highlighting a potential therapeutic direction for AEL.

## Results

### Splenomegaly precedes intrasplenic lactate accumulation in AEL model mice

**Fig. 1:**
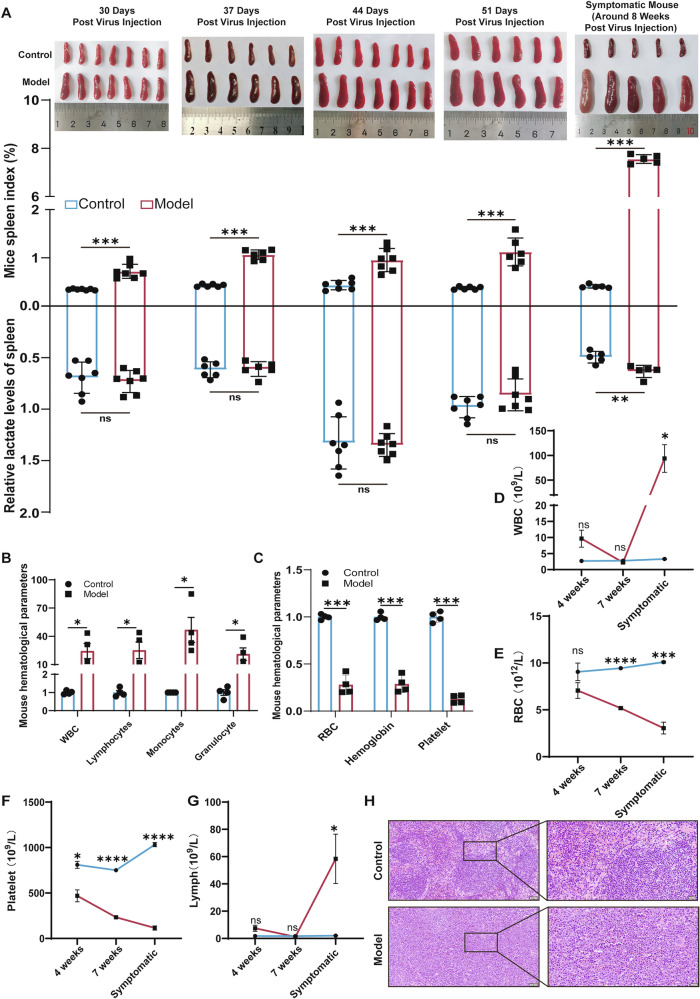
Splenomegaly precedes intrasplenic lactate accumulation in AEL model mice. **A** Representative images of spleens at indicated time points, spleen organ index (top), and the corresponding lactate content in spleen tissue are shown. *n* ≥ 5 mice per group. The proportion of the total white blood cell count (**B**) were markedly elevated, whereas red blood cell and platelet count (**C**) were significantly reduced. *n* = 4 mice per group. Changes in WBC (**D**), RBC (**E**), platelets (**F**), lymphocytes (**G**) are shown at 4 weeks, 7 weeks and upon symptom onset. *n* ≥ 3 mice per group. **H** Representative H&E-stained images of spleens from symptomatic AEL model mice are presented; *n* = 4 mice per group. Scale bars, 20 μm, 50 μm. The data are presented as the means ± SEMs. *p < 0.05, **p < 0.01, ***p < 0.001, and ****p < 0.0001; ns nonsignificant.

To investigate the metabolic mechanisms underlying splenomegaly in AEL, we established an AEL mouse model by intraperitoneally injecting neonatal Balb/c mice with F-MuLV (clone 57) within 48 h of birth (Fig. S1A). Previous studies indicate a latency period of 4–6 weeks for AEL development in F-MuLV-infected Balb/c mice [13, 29]. We therefore dynamically monitored the spleen index and splenic lactate levels to clarify the relationship between disease progression and metabolic status. The spleen index in AEL model mice was significantly elevated by 30 days post-infection (dpi) and continued to increase over time (Fig. 1A, top). Interestingly, splenic lactate content showed no significant change during the early post-infection period (30–51 dpi) but exhibited an increasing trend in the late disease stage (approximately 58–68 dpi) (Fig. 1A, bottom), indicating that lactate accumulation in the spleen is not an early event in AEL.

As the disease advanced, F-MuLV-infected mice began displaying typical signs of illness around 8 weeks post-infection, including hunched posture, piloerection, reduced activity, and pallor of the ears and tail (Fig. S1B, left). Concurrently, we observed significant splenomegaly (Fig. S1B, right), a markedly increased spleen index (Fig. S1C), and severe weight loss (Fig. S1D), mice exhibiting these features were preliminarily classified as symptomatic. Routine blood tests performed to evaluate the clinical relevance of the F-MuLV-induced AEL mouse model, revealing an elevated white blood cell (WBC) count (Fig. 1B). Significant decreases in red blood cell (RBC) count, hemoglobin (HGB) level, and platelet (PLT) count were also detected (Fig. 1C), findings consistent with the anemia symptoms observed in clinical AEL cases.

To objectively define this disease stage, we analyzed routine blood parameters at 4 weeks, 7 weeks, and the symptomatic stage post-infection. Symptomatic mice showed significantly elevated WBC, lymphocyte, and monocyte counts, alongside significantly reduced PLT, RBC, and HGB levels (Fig. 1D–G and Fig. S1E, F). From these longitudinal hematological profiles, we defined symptomatic AEL model mice according to a composite of criteria: a spleen index ≥3%, a WBC count ≥3 × 10⁹/L, a PLT count ≤200 × 10⁹/L, an RBC count ≤5 × 10¹²/L, and the presence of typical clinical signs. This endpoint was typically reached 58–68 days (8 weeks) post-infection. Unless otherwise specified, all subsequent analyses used mice at this stage and their corresponding controls. Hematoxylin and eosin (H&E) staining of spleens from symptomatic AEL mice (8 weeks post-infection) revealed complete architectural disruption with indistinct boundaries between white and red pulp (Fig. 1H). In summary, we successfully established and characterized a symptomatic F-MuLV-induced AEL mouse model, defining the symptomatic stage with multidimensional data. Lactate accumulation in the spleen occurred not as an initiating event but during the late stage, after splenomegaly was established, suggesting lactate may play an important role in AEL progression.

### Proteomic profiling reveals dysregulation of the glycolysis/gluconeogenesis axis in the spleens of AEL model mice

**Fig. 2:**
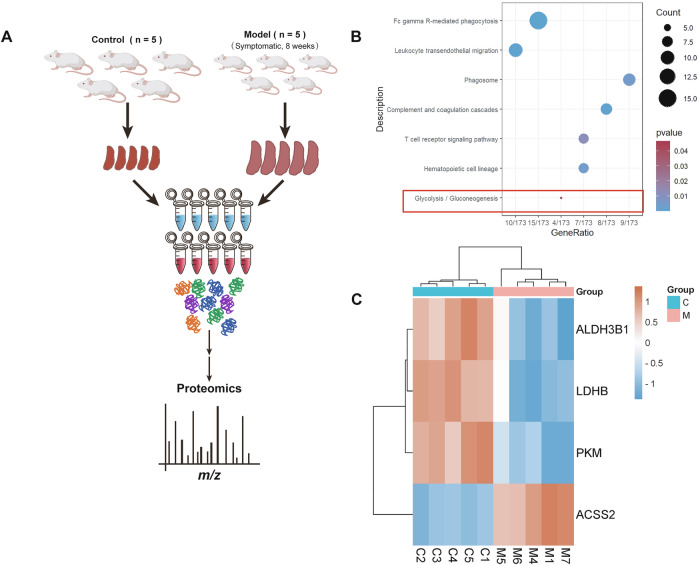
Proteomic profiling reveals dysregulation of the glycolysis/gluconeogenesis axis in the spleens of AEL model mice. **A** Schematic of the TMT proteomic analysis of spleen tissues from 10 mice, where tissues from the model group were obtained from AEL mice exhibiting symptoms. **B** KEGG enrichment analyses of differentially expressed proteins in spleen tissues from normal and symptomatic AEL model mice. **C** Four differentially expressed proteins enriched in the glycolysis/gluconeogenesis metabolic pathways; *n* = 5 mice per group. Data are presented as the means ± SEMs. *p < 0.05, **p < 0.01, ***p < 0.001, and ****p < 0.0001.

To investigate the molecular mechanisms of lactate accumulation in the spleens of symptomatic AEL model mice, we performed proteomic sequencing on the spleens of symptomatic AEL model mice (8 weeks post infection), as shown in Fig. 2A. The PCA results confirmed that the spleens of AEL model mice and normal control mice exhibited distinct profiles with good reproducibility within each group (Fig. S2A). Cluster analysis was employed to analyze the differentially expressed proteins (Fig. S2B), and a volcano plot revealed upregulated expression in 268 proteins and downregulated expression in 74 proteins (Fig. S2C). To validate the proteomic data, we conducted qPCR to analyze the changes in the expression of the top ten upregulated proteins and six randomly selected downregulated proteins. The trends observed were consistent with the proteomic sequencing results (Fig. S2D, E). GO analysis revealed enrichment of multiple biological processes, cellular components, and molecular functions related to immune cells (Fig. S2F). Additionally, KEGG pathway analysis revealed enrichment of differentially expressed proteins in immune-related signaling pathways and changes in the glycolysis/gluconeogenesis metabolic pathway (Fig. 2B). Figure 2C displays a heatmap of four differentially expressed proteins enriched in the glycolysis/gluconeogenesis pathway, all of which are associated with lactate production and metabolism [30–32]. This suggests that the observed abnormal lactate accumulation and the development of splenomegaly in AEL model mice may be linked to metabolic disorders in the spleen tissue.

### Dysregulated glucose metabolism in symptomatic AEL spleens is accompanied by elevated lactate production

**Fig. 3:**
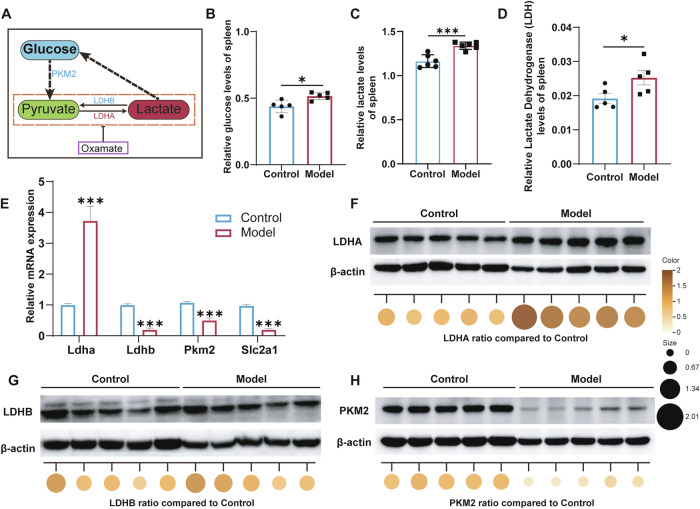
Dysregulated glucose metabolism in symptomatic AEL spleens is accompanied by elevated lactate production. **A** Modulation of lactate production through metabolic regulators. Splenic glucose (**B**) and lactate (**C**) levels were measured in symptomatic AEL model mice; *n* ≥ 5 mice per group. **D** Splenic lactate dehydrogenase (LDH) activity; *n* = 5 mice per group. **E** qPCR analysis of the mRNA expression levels of the glycolysis pathway genes *Ldha*, *Ldhb*, *PKM2*, and *Slc2a1*; *n* = 3 mice per group. **F**–**H** Western blotting and quantitative analysis of LDHA, LDHB, and PKM2 protein expression levels; *n* = 5 mice per group. The data are presented as the means ± SEMs. *p < 0.05, **p < 0.01, ***p < 0.001, and ****p < 0.0001.

Given the observed accumulation of abnormal lactate in the enlarged spleens of AEL mice during disease, alongside proteomic data revealing an imbalance in glycolytic/gluconeogenic pathways, we further evaluated the metabolic state of the splenic tissue. As depicted in Fig. 3A, lactate generation predominantly relies on the glycolytic pathway, where cytoplasmic glucose undergoes a series of catalytic reactions to form pyruvate. Therefore, we initially measured the glucose content in the spleen and observed its upregulated expression (Fig. 3B). The lactate content in the spleen of these symptomatic mice remained elevated (Fig. 3C). Assessment of pyruvate kinase (PK) activity and pyruvate content then revealed increased PK activity and decreased pyruvate content in the symptomatic AEL model mice (Fig. S3A, B). A significant increase in lactate dehydrogenase (LDH) activity was also observed (Fig. 3D). These data indicate that in symptomatic AEL model mice, elevated substrate glucose leads to pyruvate in the spleen being preferentially reduced to lactate via LDH.

To investigate the underlying molecular mechanisms, we examined changes in the expression of key glycolytic genes involved in lactate production. The results showed that lactate dehydrogenase A (LDHA), which catalyzes pyruvate conversion to lactate, was upregulated at both the transcriptional and protein levels (Fig. 3E, F). Conversely, lactate dehydrogenase B (LDHB), which catalyzes lactate oxidation, was downregulated at the mRNA level, though its protein level was unchanged (Fig. 3E, G). The expression of other key glycolytic genes, including PKM2 and SLC2A1, was also downregulated (Fig. 3E, H and Fig. S3D, E). Similarly, the expression gluconeogenesis genes (*G6pc1*, *Fbp1*, and *Pck1*), which catabolize lactate into glucose, was also reduced (Fig. S3C). These data suggest coordinated suppression of lactate catabolism alongside enhanced anabolic activity, ultimately promoting lactate accumulation. The coordinated activity of monocarboxylate transporters 1–4 (MCT1–4) is essential for maintaining lactate homeostasis and facilitating the lactate shuttle between glycolytic and oxidative cells [25]. Therefore, we examined alterations in MCT1 and MCT4 in the symptomatic AEL model mice. Western blotting analysis revealed upregulated expression of MCT1 and Hif-1α, with no change in MCT4 expression (Fig. S3F–H). This finding suggests enhanced lactate uptake coupled with reduced clearance in the enlarged spleen, indicating disrupted lactate shuttle dynamics.

Collectively, our findings implicate metabolic disorders as the underlying cause of elevated lactate levels in the AEL spleen. Specifically, high LDHA expression promotes the conversion of pyruvate to lactate. Additionally, the inhibition of gluconeogenesis impedes lactate decomposition. Dysregulated MCT1 and MCT4 expression contributes to lactate metabolism imbalance, ultimately promoting lactate uptake and accumulation in the enlarged spleen.

### Upregulation of lactylation in the spleen and macrophages of symptomatic AEL model mice

**Fig. 4:**
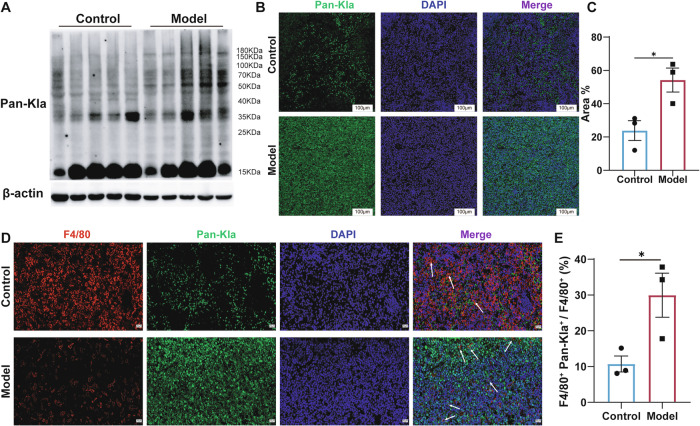
Upregulation of lactylation in the spleen and macrophages of symptomatic AEL model mice. **A** Western blotting analysis of Pan-Kla expression levels in the spleens of mice with symptomatic AEL; *n* = 5 mice per group. **B** Representative images of DAPI and Pan-Kla immunofluorescence staining of spleens from symptomatic mice, and quantitative analysis of (**C**); *n* = 3 mice per group. Scale bars, 100 μm. Representative images of F4/80 co-stained with Pan-Kla in the spleens of control and AEL model mice (**D**), and quantification of F4/80 and Pan-Kla immunoreactivity in (**E**); *n* = 3 mice per group. Scale bars, 20 μm. The data are presented as the means ± SEMs. *p < 0.05, **p < 0.01, ***p < 0.001, and ****p < 0.0001.

These results indicate that significant metabolic reprogramming occurs in the spleens of AEL model mice during the disease phase, leading to aberrant lactate production and accumulation. Lactate is known to function as an important signaling molecule that can directly regulate cellular functions, such as macrophage polarization, through protein lactylation [26]. We therefore hypothesized that the abnormal lactate accumulation in the spleens of symptomatic AEL model mice might influence the function of their resident immune cells. To test this, we assessed spleen tissues using a pan-lactylation (Pan-Kla) antibody. The analysis revealed a significant increase in overall protein lactylation levels in the spleen tissues of AEL model mice during the disease phase (Fig. 4A). This finding was corroborated by immunofluorescence staining (Fig. 4B, C). Macrophages are essential for tissue repair and immunity, these cells differentiate into activated/pro-inflammatory macrophages (M1) or alternatively activated/anti-inflammatory macrophages (M2) [33]. M1 macrophages are characterized by lactate production and increased histone Kla, whereas M2 macrophages exhibit increased oxidative phosphorylation and fatty acid oxidation [26, 34]. We therefore evaluated the immunofluorescence co-localization of F4/80, a macrophage marker, and Pan-Kla antibody. A strong lactylation fluorescence signal was spatially co-localized with F4/80⁺ macrophages (Fig. 4D, E), indicating significant lactylation modification within splenic macrophages in the lactate-accumulating microenvironment. These results demonstrate that the lactate-accumulating microenvironment in symptomatic AEL model spleens induced extensive protein lactylation, which was also markedly expressed in macrophages. This suggests that the function and state of immune cells within the splenic tissue may be remodeled by this high-lactate microenvironment.

### Immune cell remodeling and macrophage polarization towards the M1 phenotype in the spleens of symptomatic AEL model mice

**Fig. 5:**
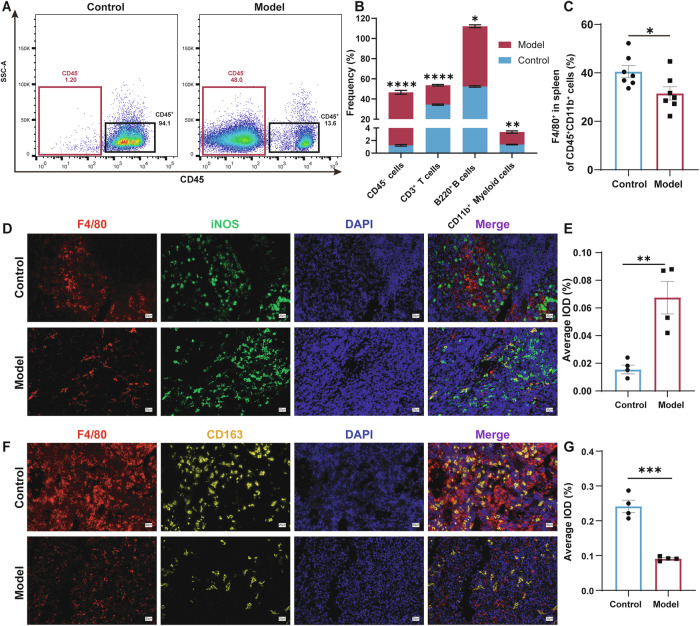
Immune cell remodeling and macrophage polarization towards the M1 phenotype in the spleens of symptomatic AEL model mice. **A** Representative flow cytometry dot plots depict CD45 expression, with CD45^-^ and CD45^+^ cell populations gated in red and black boxes, respectively. **B** Quantitative proportions of CD45^-^ cells, T cells, B cells, and myeloid cells in the spleen. **C** Flow cytometry analysis of macrophages (CD11b^+^F4/80^+^); these cells were isolated from the spleens of control and symptomatic AEL model mice; *n* = 7 mice per group. Representative images of F4/80 co-stained with iNOS in the spleens of control and symptomatic AEL model mice (**D**), and quantification of F4/80 and iNOS immunoreactivity in (**E**); *n* = 3 mice per group. Scale bars, 20 μm. Representative images of F4/80 co-stained with CD163 in the spleens of control and symptomatic AEL model mice (**F**), and quantification of F4/80 and CD163 immunoreactivity in (**G**); *n* = 3 mice per group. Scale bars, 20:μm. The data are presented as the means ± SEMs. *p < 0.05, **p < 0.01, ***p < 0.001, and ****p < 0.0001.

As the largest secondary lymphoid organ, the spleen performs extensive immune functions [35]. We previously observed that macrophages in the splenic high-lactate microenvironment exhibit extensive lactylation. To delineate the immunological consequences of splenomegaly in AEL, we performed flow cytometric characterization of splenic immune populations in symptomatic AEL mice (Fig. S4A). Relative to spleens from normal controls, the proportion of CD45⁺ cells in the model group decreased by approximately 78%, whereas non-immune CD45⁻ cells increased by about 35% (Fig. 5A and Fig. S4B, C). Lymphocyte composition was also altered, with a 44% reduction in CD3⁺ T cells and a 14% increase in B cells, while CD11b⁺ myeloid cells were upregulated by 46% (Fig. 5B and Fig. S4D, E). Further subset analysis revealed a selective 16% decrease in CD4⁺ helper T cells, concurrent with a 39% expansion of the CD8⁺ cytotoxic T lymphocyte population (Fig. S4F). Moreover, the average proportion of CD11b⁺CD11c⁺ cells increased from 7.13 to 27.97% (Fig. S4G). These findings demonstrate an imbalance in immune cell subset proportions within the spleens of AEL mice.

Furthermore, we observed quantitative and qualitative changes in the splenic populations. The total number of F4/80⁺ macrophages decreased by 8% (Fig. 5C), which was corroborated by immunohistochemical quantification, which revealed a 32% reduction in the cellular density (Fig. S4H, I). Polarization analysis revealed profound M1-skewed differentiation: the number of iNOS⁺ M1 macrophages increased 4.4-fold (Fig. 5D, E), whereas that of their CD163⁺ M2 counterparts decreased by 62% (Fig. 5F, G). Collectively, these findings demonstrate that the enlarged spleens of AEL model mice exhibited reduced macrophage expression accompanied by an imbalance in M1/M2 polarization, suggesting that alterations in the immune response of the tumor microenvironment and their impact on macrophage polarization may enhance proinflammatory responses. In summary, symptomatic AEL model mice exhibited an imbalance in splenic immune cell proportions driven by metabolic reprogramming and abnormal lactate accumulation. T lymphocytes were generally downregulated, while B cells and myeloid cells were upregulated. Concurrently, the total number of macrophages decreased and their phenotype shifted toward M1 polarization.

### Mechanistic link between dual STAT1–mTOR signaling and lactate-mediated M1 polarization in macrophages

**Fig. 6:**
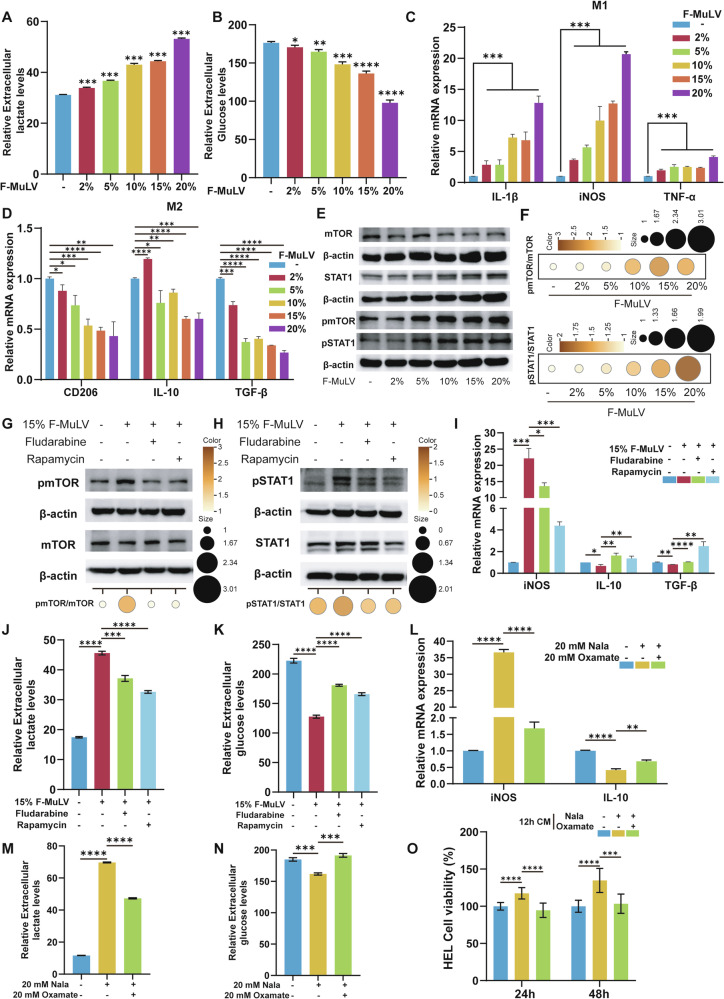
Mechanistic link between dual STAT1–mTOR signaling and lactate-induced M1 polarization in macrophages. Detection of lactate (**A**) and glucose (**B**) levels in cell supernatants after treating RAW264.7 macrophages with different concentrations of F-MuLV for 24 h; *n* = 3. **C, D** qPCR analysis of the mRNA expression levels of *IL-1β*, *iNOS*, *TNF-α*, *CD206*, *IL-10*, and *TGF-β* in RAW264.7 macrophages treated with different concentrations of F-MuLV for 24 h. **E**, **F** Western blotting and quantitative analysis of the protein expression levels of STAT1, pSTAT1, mTOR, and pmTOR in macrophages treated with different concentration of F-MuLV for 24 h. **G**, **H** Western blotting and quantitative analysis of the protein expression levels of STAT1, pSTAT1, mTOR, and pmTOR in macrophages treated with 15% F-MuLV combined with the STAT1 inhibitor Fludarabine (20 μM) or the mTOR inhibitor Rapamycin (100 nM) for 24 h. **I**–**K** qPCR analysis measured *iNOS*, *IL-10*, and *TGF-β* mRNA expression in RAW264.7 macrophages treated with 15% F-MuLV and the indicated inhibitors for 6 h (**I**). Lactate (**J**) and glucose (**K**) levels in the corresponding supernatants are shown. **L**–**N** qPCR analysis evaluated *iNOS* and *IL-10* mRNA expression in RAW264.7 macrophages treated with 20 mM Nala with or without 20 mM Oxamate for 12 h (**L**). Lactate (**M**) and glucose (**N**) levels in the corresponding cell supernatants are shown. **O** Conditioned medium (CM) from RAW264.7 macrophages treated with 20 mM Nala with or without 20 mM Oxamate for 12 h was assessed for its effect on the growth of HEL cells after 24 and 48 h of incubation. *n* = 3. The data are presented as the means ± SDs. *p < 0.05, **p < 0.01, ***p < 0.001, and ****p < 0.0001.

To further explore the regulatory mechanism of F-MuLV on macrophages in vitro, we incubated RAW264.7 cells with different concentrations of F-MuLV for 24 h to mimic the macrophages in AEL model mice. With increasing concentrations of F-MuLV, macrophages exhibited morphological characteristics of M1 polarization [36], while their growth was further inhibited (Fig. S5A, B). Figure 6A and B show that after 24 h of F-MuLV infection, lactate levels in the cell supernatant increased significantly, while glucose levels decreased, both in a dose-dependent manner (Fig. 6A, B). Furthermore, qPCR analysis revealed that the expression levels of the M1 macrophage marker genes *IL-1β*, *iNOS*, and *TNF-α* upregulated with increasing F-MuLV concentrations, whereas the expression of the M2 macrophage marker genes *CD206*, *IL-10*, and *TGF-β* downregulated (Fig. 6C, D). These data indicate that F-MuLV-infected macrophages preferentially polarize toward the M1 phenotype, mirroring observations in the enlarged spleens of AEL model mice. Western blotting analysis of infected macrophages revealed that F-MuLV can activate the NF-κB, STAT1, and mTOR signaling pathways (Fig. 6E, F and Fig. S5C, D).

To verify the necessity of these pathways, RAW264.7 cells were infected with 15% F-MuLV in the presence of the specific inhibitors Fludarabine (targeting STAT1) and Rapamycin (targeting mTOR). Inhibition of either signaling pathway (Fig. 6G, H) blocked the virus-mediated increase in lactate production and the upregulation of the M1 gene *iNOS* (Fig. 6I). This treatment also restored glucose levels and elevated expression of the M2 genes *IL-10* and *TGF-β* (Fig. 6I–K). These results indicate that STAT1 and mTOR activation acts as a critical molecular switch for F-MuLV-driven metabolic reprogramming in macrophages.

To examine the effect of lactate on macrophage function, we treated macrophages with sodium lactate (Nala) at different concentrations. Treatment for 6, 12, and 24 h significantly increased the expression of *iNOS*, which peaked at 12 h (Fig. S5E), and elevated lactate levels in the supernatant (Fig. S5H). This treatment also suppressed *IL-10* and *TGF-β* expression (Fig. S5F, G) while reducing supernatant glucose concentrations (Fig. S5I). The combination of the lactate production inhibitor sodium oxamate (Oxamate, 20 mM) with a high concentration of Nala (20 mM) reversed these changes (Fig. 6L–N), demonstrating that lactate promotes M1 macrophage polarization. Moreover, a low concentration of Nala (10 mM) combined with 15% F-MuLV further enhanced *iNOS* expression and supernatant lactate content. Under these conditions, IL-10 and *TGF-β* expression and glucose levels were also suppressed (Fig. S5J–L). These results indicate a synergistic interaction between the virus and lactate in driving M1 polarization and metabolic reprogramming in macrophages.

To determine whether macrophage functional alterations in a high-lactate environment promote leukemia progression, we collected conditioned media (CM) from macrophages treated with Nala alone or with Oxamate for 12 h and used it to treat human erythroleukemia HEL cells. CM from the Nala-treated group significantly enhanced HEL cell proliferation at 24 and 48 h, an effect that was blocked by co-treatment with Oxamate (Fig. 6Q). This indicates that the macrophage niche under high-lactate conditions supports leukemia cell proliferation.

In summary, these findings demonstrate that lactate is not merely a product of STAT1/mTOR-mediated metabolic reprogramming and M1 polarization in macrophages. Instead, it actively reinforces their activated phenotype and shapes a cellular niche that promotes leukemia cell proliferation.

### In vivo lactate depletion attenuates splenomegaly and improves survival in AEL model mice

**Fig. 7:**
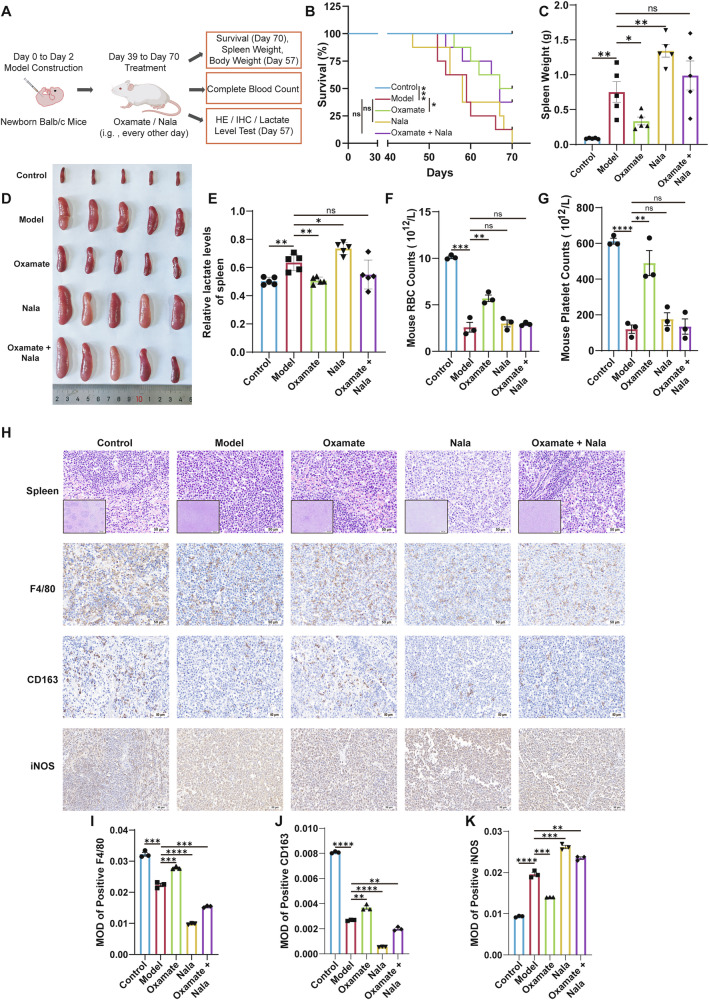
In vivo lactate depletion attenuated splenomegaly and improved survival in AEL model mice. **A** Schematic of the treatment strategy for erythroleukemia model mice with the lactate production inhibitors Oxamate (500 mg/kg) and Nala (500 mg/kg) and their combination. **B** Survival curves of the mice in the control, model, Oxamate, Nala, and Oxamate + Nala groups during treatment; *n* = 8 mice per group. Weight (**C**) and appearance (**D**) of spleens from each group of mice; *n* = 5 mice per group. **E** Lactate content in the spleens of each group. **F**, **G** Routine blood examination of each group; *n* = 3 mice per group. **H** Representative images of H&E, F4/80, CD163 and iNOS IHC staining of spleen tissues from each group, with quantitative analysis of F4/80 in (**I**), CD163 in (**J**) and iNOS in (**K**); *n* = 3 mice per group. Scale bars, 500 μm, 50 μm. The data are presented as the means ± SEMs. *p < 0.05, **p < 0.01, ***p < 0.001, and ****p < 0.0001; ns nonsignificant.

To further investigate the role of lactate in splenomegaly in AEL model mice, we examined the impacts of blocking lactate production in vivo on mouse spleen size and survival duration. Figure 7A illustrates the experimental protocol for in vivo lactate blockade. Following 39 days of neonatal mouse modeling, the animals were randomly assigned to groups and received gavage at 2-day intervals. As depicted in Fig. S6A, notable differences in body weight emerged after the initial administration, but these differences did not persist over time. Survival analysis showed that the treatment group, which received the lactate production inhibitor Oxamate, exhibited significantly prolonged survival compared to the model group, with median survival times of 68.5 days and 59 days, respectively. The median survival times for the Nala group and the combination group were 58 days and 67 days. These findings demonstrate that inhibiting lactate production in vivo effectively extends survival in this AEL mouse model (Fig. 7B).

When symptoms became apparent on day 57 of administration, we euthanized five randomly selected mice from each group via deep anesthesia and dissection to assess visceral organ changes. Our findings revealed that the liver, spleen, and lungs of the mice in the Oxamate group were significantly smaller and lighter than those in the AEL model group (Fig. S6B, C). Conversely, the spleen size and weight were greater in the Nala administration group than in the AEL model group (Fig. 7C, D). The lactate content in the spleen was reduced in the Oxamate group and elevated in the Nala administration group (Fig. 7E). Blood analysis indicated that inhibiting lactate production in vivo significantly increased the levels of RBC, PLT, and HGB (Fig. 7F, G and Fig. S6D), while reducing WBC, lymphocyte, neutrophil, and monocyte count in the Oxamate group (Fig. S6E). These changes suggesting that suppressing lactate generation can notably improve peripheral blood parameters in AEL model mice.

H&E staining revealed that Oxamate treatment mitigated pathological structural disorganization not only in the spleen but also in extramedullary organs, including the liver, lungs, and kidneys of AEL mice (Fig. 7H and Fig. S6F). In line with the proposed mechanism, immunohistochemistry of spleen sections indicated that Oxamate administration substantially reversed the disease-associated dysregulation of macrophages, elevating expression of the M2 marker CD163 while reducing levels of the M1 marker iNOS (Fig. 7H). Quantitative IHC scoring confirmed these phenotypic changes (Fig. 7I–K). Collectively, our in vivo intervention data indicate that pharmacologically inhibiting lactate production alleviates splenomegaly, prolongs survival, and corrects the pathogenic M1-polarized macrophage imbalance in the AEL microenvironment. These results identify lactate as a critical disease-promoting metabolite and support targeting the lactate-driven immunometabolic axis as a potential adjunctive therapeutic strategy for AEL.

## Discussion

Splenomegaly in AML results from extensive leukemic blast infiltration and correlates with poor prognosis, yet the pathogenic mechanisms driving this process remain poorly understood [37, 38]. The specific mechanisms underlying splenomegaly in AEL, a distinct AML subtype, are similarly unclear. Using a classic F-MuLV-induced AEL model, this study investigated the critical changes occurring in the spleen during disease establishment and progression.

In this study, significant lactate accumulation was observed in splenic tissue during the advanced stage of AEL, when mice exhibited symptomatic disease. Through gain‑of‑function and loss‑of‑function experiments, lactate was causally implicated in disease progression: exogenous lactate treatment alone induced M1 polarization of macrophages. Conversely, in vivo blockade of lactate production with Oxamate reversed the polarization state of splenic macrophages, downregulating iNOS and upregulating CD163, and also alleviated splenomegaly while prolonging survival. These findings establish the lactate‑macrophage M1 polarization axis as a key driver of advanced AEL progression, suggesting its therapeutic relevance. Furthermore, elevated LDH activity was observed, accompanied by upregulated LDHA and downregulated LDHB expression, providing an enzymatic basis for the splenic lactate accumulation [39, 40].

Mechanistically, F-MuLV infection was shown to activate the STAT1/mTOR signaling pathway in macrophages. Inhibiting either STAT1 or mTOR activation concurrently reduced both lactate production and the expression of M1 polarization markers. Lactate itself was identified not as a simple metabolic byproduct but as an important immunomodulatory signal that directly reshapes macrophage functional phenotype. This interaction establishes a self-reinforcing cycle wherein lactate promotes macrophage M1 polarization, and the enhanced glycolysis of M1 macrophages produces more lactate [36]. This increased lactate accumulation further stabilizes the M1-polarized state. The cycle likely represents a core mechanism that drives accelerated disease progression in advanced stages.

The lactate-driven M1 polarization of macrophages must be considered within the broader context of immune microenvironment remodeling in the spleen during AEL. In symptomatic AEL model mice, the proportion of CD45⁺ leukocytes in splenic tissue decreased from a median of 94.66% to 20.61%, while CD45⁻ non-immune cells, likely erythroid precursors, increased significantly from 1.244 to 45.37%. This shift demonstrates both the malignant clonal expansion that characterizes the disease and a concurrent remodeling of the immune microenvironment. Lactate and LDHA are established contributors to immunosuppression, as extensive research confirms that lactate production driven by LDHA can impair T cell and natural killer (NK) cell anti-tumor functions while promoting the recruitment of myeloid-derived suppressor cells (MDSCs), thereby weakening immune surveillance [41–44]. We observed a slight upregulation in the CD11b⁺ myeloid cell population, alongside a decrease in the CD11b⁺F4/80⁺ macrophage subset and an upregulation of CD11b⁺CD11c⁺ cells. These findings suggest an imbalance in immune homeostasis in AEL mice, dominated by malignant cells and accompanied by the expansion of myeloid subsets like dendritic cells (DCs) and an impairment of lymphocytes. In solid tumors, lactate is known to induce macrophages to express M2-associated genes such as *VEGF* and *Arg1*, promoting angiogenesis and immunosuppression [45]. Furthermore, myeloid-specific LDHA knockout, which eliminates cell-autonomous lactate production, promotes macrophage reprogramming toward an M1 phenotype and inhibits tumor growth [46]. In contrast, our study found that inhibiting lactate generation in AEL spleen tissues reversed macrophage polarization toward the M2 phenotype. This discrepancy indicates that lactate functions as a multifaceted immune modulator, underscoring how distinct metabolic programs and the overall metabolic state of the microenvironment differentially influence immune cell fate [33, 46].

This study has several limitations that also highlight avenues for future work. First, temporal dynamics analysis revealed that splenic lactate levels became elevated only after splenomegaly was established, suggesting that lactate drives disease progression rather than initiates it. Second, while inhibiting lactate production with Oxamate primarily remodels the immune microenvironment, its effect on the cell intrinsic malignant potential of leukemia cells, such as their regenerative capacity after in vivo transplantation, remains unclear. Addressing this question is a critical next step to assess whether such an adjuvant strategy could contribute to disease eradication. Third, whether this approach is specific to AEL or extends to other forms of splenomegaly requires validation using non-malignant splenomegaly control models. Additionally, the precise cellular source of lactate in the spleen and its intercellular shuttling via MCT1/MCT4 warrant further investigation using lineage tracing techniques [47–50]. Fourth, validating the mechanism by which lactate regulates polarization via pathways including STAT1/mTOR in macrophage‑conditional gene knock‑out models would yield more direct genetic evidence. Finally, evaluating the synergistic efficacy of targeted lactate metabolism intervention combined with anti-AEL drugs represents a clear and promising direction.

In summary, using an F-MuLV induced AEL mouse model, we found that abnormal lactate accumulation in the enlarged spleens of symptomatic mice drives macrophage M1 polarization during late-stage disease. We further demonstrate that AEL associated splenomegaly is not merely a passive cellular accumulation of cells, but is actively sustained by a self-reinforcing cycle between lactate and M1 macrophages. This work provides a novel metabolic-immunological perspective on the pathophysiology of leukemic splenomegaly and suggests that targeting this cycle may represent an effective adjuvant therapeutic strategy for AEL and other hematologic malignancies with splenomegaly.

## Materials and methods

### Cell Culture and in vitro Inhibitor/Stimulation Assays

Murine RAW264.7 macrophages, NIH-3T3 cells containing F-MuLV clone 57 and human erythroleukemia (HEL) cells, were cultured in DMEM supplemented with penicillin (100 U/mL), streptomycin (100 ng/mL), and 10% FBS. All the cells were incubated at 37 °C with 5% CO_2_.

The macrophages were treated with the F-MuLV mixture at concentrations of 2%, 5%, 10%, 15%, or 20% for 24 h. In pathway inhibition experiments, RAW264.7 cells were pre-treated with 20 µM Fludarabine (MCE) or 100 nM Rapamycin (MCE) for 3 h prior to a 6-h co-treatment with 15% F-MuLV. To assess lactate function, RAW264.7 cells were treated with Nala (10 mM, 20 mM; Sigma) for 6, 12, or 24 h. For combination treatments, cells were incubated with 15% F-MuLV and 10 mM Oxamate (MCE) for 12 h, or with 20 mM Nala and 20 mM Oxamate (MCE) for 12 h.

### Preparation of Conditioned Medium (CM)

RAW264.7 cells were treated for 12 h with 20 mM Nala, either with or without 20 mM Oxamate. The cell culture supernatant was collected, centrifuged at 2500 rpm for 10 min at room temperature, and filtered through a 0.45 μm membrane. This prepared CM was stored at −20 °C. Prior to use, it was thawed, mixed 1:1 with fresh medium, and then co-cultured with target cells for the indicated time.

### Cell Viability

RAW264.7 cells (10,000 cells/well) and HEL cells (7000 cells/well) were seeded in 96-well plates in 100 μL of medium. After 24 h, the medium for RAW264.7 cells was replaced with medium containing various concentrations of F-MuLV, and incubation continued for a further 24 h. For HEL cells, the medium was replaced with a 1:1 mixture of fresh and CM, followed by incubation for 24 or 48 h. Then, 10 μL of MTT solution (5 mg/mL, Solarbio) was added to each well, and the plates were incubated at 37 °C for 4 h. After carefully removing the supernatant, 100 μL of DMSO was added to each well to solubilize the formazan crystals for viability determination.

### Preparation of viral supernatant

The F-MuLV clone 57 strain (devoid of SFFV [51, 52]) was a kindly provided by Professor Yaacov Ben-David. NIH-3T3 cells harboring this strain were cultured in serum-free DMEM for 48 h to produce a viral supernatant. This supernatant was centrifuged at 2500 rpm for 15 min to remove cellular debris. The clarified supernatant was then filtered through a 0.45 μm membrane. Filtered viral supernatant was aliquoted and stored at −80 °C. For use, aliquots were thawed on ice.

### Leukemia induction and in vivo drug studies

Female and male Balb/c mice were purchased from Beijing HFK Bioscienc. Neonatal Balb/c mice within 48 h of birth received an intraperitoneal (i.p.) inoculation of 100 μL of viral supernatant (3000 focus-forming units) using a 1-cc U-100 insulin syringe, as described previously [52, 53].

Mice were grouped 3–4 weeks after viral injection. Beginning on day 39 post-infection, mice were administered Oxamate (0.5 g/kg body weight), Nala (0.5 g/kg body weight), a combination of both, or a control (0.9% NaCl) by oral gavage (i.g.) every other day. The mice were sacrificed using CO_2_ after 18 days of treatment. The organs, including the heart, liver, spleen, lungs, and kidneys, were removed, photographed and weighed for organ index (organ weight/body weight) calculation. The specimens were fixed with formalin solution, then sectioned, and stained.

### Proteomics

The samples were dissolved in lysis buffer, ground with three titanium dioxide abrasive beads, and concentrated at 5000 × *g* for 5 min at 4 °C. The supernatant was collected and centrifuged at 15,000 × *g* for 30 min at 4 °C. The final supernatant was collected and stored at −80 °C until use. The total protein concentration was measured using the Bradford protein assay. Next, the proteins were subjected to enzymatic hydrolysis for desalination, and the proteins were labeled with TMT reagents. Finally, the samples were analyzed using LC‒MS/MS mass spectrometry [54].

### RNA isolation and real-time quantitative PCR assays (qPCR)

Total RNA was extracted from mouse spleens and RAW264.7 cells using TRIzol reagent. According to the manufacturer’s instructions, complementary DNA was synthesized using a FastKing RT kit (with gDNase). qPCR was conducted using the SYBR Green Premix Pro Taq HS qPCR Kit. Gene-specific primers with the sequences listed in Table S1 were used for the qPCR assay. β-Actin was used as a housekeeping control. The relative gene expression levels were analyzed using the 2^–△△CT^ method [55].

### Flow cytometry cell analysis

For single-cell suspension preparation, mouse spleens were isolated and cut into small pieces on ice, followed by grinding through a 70-μm cell strainer (SPL). The resulting homogenate was then extracted using a 1 mL syringe with a 27-gauge needle. The cells were washed twice with PBS. Then, the red blood cells were lysed with red blood cell lysis buffer. The dead cells were stained with the Zombie NIRTM Fixable Viability Kit (BioLegend) for 15 min at room temperature in the dark.

The cells were subsequently washed with FACS buffer (PBS supplemented with 1% FBS). Phenotype analysis was performed through incubation with the following antibodies purchased from BioLegend on ice for 30 min in the dark: anti-CD45-PerCp/Cyanine5.5 (S18009F), anti-CD3-FITC (17A2), anti-CD4-APC (RM4-4), anti-CD8-BV510TM (53-6.7), anti-CD19-BV421 (6D5), anti-B220-PE (RA3-6B2), anti-CD11b-BV605 (M1/70), anti-CD11c-APC (N418), and anti-F4/80-PE (BM8). Antibodies listed in Table S2. The detailed gating strategy for flow cytometry is presented in Fig. 4A. Flow cytometry was performed using a FACS instrument and FlowJo software.

### Western blotting

Western blotting was performed according to the standard protocol. Briefly, the protein was extracted using RIPA lysis buffer supplemented with PMSF and phosphatase inhibitors. Following centrifugation at 12,000 rpm for 15 min at 4 °C, the protein concentrations in the extracts were quantified using a BCA assay kit. SDS‒PAGE gels (10%) were prepared and used to separate proteins, which were subsequently transferred from the gel to PVDF membranes. The membranes were blocked for 1.5 h at room temperature in 5% BSA solution. Subsequently, the membranes were incubated overnight at 4 °C with the appropriate primary antibody, followed by incubation with the secondary antibody. Antibodies listed in Table S2. For cells, each experiment included three independent biological replicates. Target band intensities were quantified using ImageJ software. For each lane, the signal intensity of the target protein was normalized to that of β-actin as an internal reference. The final value for each condition represents the arithmetic mean of the three normalized replicates. In the figures, these data are displayed as bubble plots, where bubble size and color denote the mean normalized expression level.

### Immunofluorescence staining

Symptomatic AEL model spleen and normal control mouse spleens were immediately placed in 4% paraformaldehyde fixative and incubated for at least 24 h at room temperature. The fixed tissues were dehydrated, paraffin-embedded, and subsequently cut into 4-μm-thick sections using a microtome. The paraffin-embedded sections were washed three times with PBS and blocked with blocking buffer (10% rabbit serum or 3% BSA in PBS) at room temperature for 30 min, followed by an overnight incubation with primary antibodies at 4 °C. After being washed with PBS three times, the sections were incubated with species-matched HRP-labeled secondary antibodies at room temperature for 50 min. Following washing with PBS, the sections were incubated with the corresponding TSA reagents at room temperature for 10 min in the dark. The tissue sections were placed in citrate-based antigen retrieval solution and heated in a microwave oven for 10 min for boiling to remove the bound primary/secondary antibodies. The subsequent steps, including serum-blocking primary/secondary antibody incubation and TSA incubation, were then repeated. Nuclei were stained with DAPI. Finally, observations were made under an upright fluorescence microscope (NIKON ECLIPSE C1), and immunofluorescence images were acquired using a scanner (Pannoramic).

### Immunohistochemistry (IHC)

The paraffin sections were dewaxed and rehydrated, and antigen retrieval was performed. The sections were immersed in 3% hydrogen peroxide and incubated at room temperature in the dark for 25 min to block endogenous peroxidase activity. Following three washes with PBS, the samples were blocked with 3% BSA blocking buffer at room temperature for 30 min. The samples were blocked with primary antibody at 4 °C overnight and incubated with HRP-labeled secondary antibody at room temperature for 50 min, followed by color development using freshly prepared DAB chromogen solution. Nuclei were counterstained with hematoxylin. Slides were imaged using a fluorescence microscope (Nikon). The staining intensity was graded using ImageJ as follows: 0, no staining; 1, weak staining (light yellow); 2, moderate staining (yellowish-brown); and 3, strong staining (brown).

### H&E

The paraffin sections were dewaxed, rehydrated, treated with high-definition constant staining pretreatment solution for 1 min, stained with hematoxylin for 3–5 min, and then sequentially processed through the following cycle: water rinsing→differentiation solution→water rinsing→bluing solution→water rinsing. The sections were dehydrated in 95% ethanol for 1 min and stained with eosin for 15 s. Observation was performed under an upright fluorescence microscope (Nikon), and images were captured using an imaging system (Nikon). The results revealed blue-stained nuclei and red-stained cytoplasm.

### Measurement of pyruvate, pyruvate kinase, LDH, lactate and glucose levels

Spleen samples were collected from the AEL model and the control groups and then preserved in a − 80 °C freezer. Prior to use, mouse spleen tissues were sectioned into small fragments and weighed. 0.9% saline was added at a tissue-to-saline ratio of 1:9 for homogenization, followed by centrifugation at 2500 rpm for 10 min at 4 °C. The supernatant was collected for quantitative detection of LDH, pyruvate kinase, pyruvate, lactate and glucose levels according to the manufacturer’s protocol. For the cellular samples, the cell culture supernatant was collected and centrifuged at 4 °C and 2500 rpm for 10 min. After dilution to appropriate concentrations, glucose and lactate levels in the supernatant were measured following the manufacturer’s instructions.

### Survival and statistical analysis

Statistical analysis was performed using GraphPad Prism software 8.3.0. Two groups were compared using Student’s *t* test (two-sided). Survival differences were determined using the Kaplan‒Meier method and log-rank test. The data are expressed as the means ± SDs or means ± SEMs. The statistical details and sample sizes (n) can be found in the figure legends. Differences are represented by *p* values. Differences were considered significant at a *p* < 0.05.

*p < 0.05, **p < 0.01, ***p < 0.001, ****p < 0.0001, and ns: nonsignificant.

## Supplementary information

SUPPLEMENTAL MATERIAL

Original Western blots

## Data Availability

All data are available in the main text or in supplemental material. Supplemental material includes supplemental figures, methods, tables. For original data, please contact the corresponding author.
