# Supplementary material for: Lactate-mediated macrophage polarization promotes splenomegaly in acute erythroleukemia
Source: Cell Death Dis. 2026 Mar 25;17(1):373. doi: 10.1038/s41419-026-08612-5 (PMC13039512; doi:10.1038/s41419-026-08612-5)
Supplement: Supplementary file 1 — SUPPLEMENTAL MATERIAL [file 41419_2026_8612_MOESM1_ESM.docx]

**Lactate-mediated macrophage polarization promotes splenomegaly in acute erythroleukemia**

**Supplemental Materials**

**Mingyue Yang^1,2,3,4^, Dan Xie^1,2,3,4^, Yanlong Zhang^1^, Yi Ye^2,4^, Suwen Yang^4^, Hongqian Zhu^5^, Sha Cheng^2,4^, Jia Yu^2,4^, Ningning Zan^2,4^, Shengwen Huang^*1,3^ and Heng Luo^*1,2,4^**

**Institutional Affiliations:**

^1^Guizhou University Medical College, Guiyang 550025, China.

^2^State Key Laboratory of Discovery and Utilization of Functional Components in Traditional Chinese Medicine, Guizhou Medical University, Guiyang 550014, China.

^3^Department of Medical Genetics, Guizhou Provincial People’s Hospital, Guiyang 550499, China.

^4^Natural Products Research Center of Guizhou Province, Guiyang 550014, China.

^5^Department of Haematology, Guizhou Provincial People's Hospital, Guiyang 550499, China.

**Supplemental Tables:**

**Supplemental Table 1 qPCR primers used in this paper**

| Gene name | Forward | Reverse |
| --- | --- | --- |
| TNFα | CAGGCGGTGCCTATGTCTC | CGATCACCCCGAAGTTCAGTAG |
| IL-1β | GCAACTGTTCCTGAACTCAACT | ATCTTTTGGGGTCCGTCAACT |
| iNOS | GTTCTCAGCCCAACAATACAAGA | GTGGACGGGTCGATGTCAC |
| CD206 | CTCTGTTCAGCTATTGGACGC | CGGAATTTCTGGGATTCAGCTTC |
| IL-10 | GCTCTTACTGACTGGCATGAG | CGCAGCTCTAGGAGCATGTG |
| TGFβ | TCTGCATTGCACTTATGCTGA | AAAGGGCGATCTAGTGATGGA |
| Fhl1 | GACTGCCGCAAGCCCATAA | CCAAGGGGTGAAGGCACTT |
| Fgg | ACCAGAGATAACTGTTGCATCCT | CCACGTCGGTTTGGTAAGAAG |
| Blk | GAGGCAGGTCAGTGAGAAGG | GTCCTGGTTAGGAGATGGTGG |
| Syk | CTACCTGCTACGCCAGAGC | GCCATTAAGTTCCCTCTCGATG |
| Icam1 | GTGATGCTCAGGTATCCATCCA | CACAGTTCTCAAAGCACAGCG |
| Rftn1 | ATGGGTTGCAGTTTGAACAAGC | GTCACATCTACTTTGGTCTCCAC |
| PKM2 | GCCGCCTGGACATTGACTC | CCATGAGAGAAATTCAGCCGAG |
| Hdgf | CCGGATTGATGAGATGCCTGA | TTGCCAAACTTCTCCTTGGATT |
| Ccdc25 | CAGCAGCAGCGTTAATTCATC | GGCCGAAGAGAGTTTGTCCAC |
| Enpp4 | GTCACTGGCTATAAAGGGAACTC | TTCCACCAAGACACCTTCTTTG |
| Pafah2 | GGAGGGTCACAGTCTTGAGG | CCCATGCTGTACTCGTAGCG |
| Uck2 | CTTCCGTTTGTGCTAAGATCGT | GTGAGGACTCGGTAGAAGCTAT |
| Car1 | GACTGGGGATATGGAAGCGAA | TGCAGGATTATAGGAGATGCTGA |
| Samsn1 | TTCACGCCAAGTCCCTATGAC | TTCCCATTGGTGTTTTGCAGATA |
| CDK6 | GGCGTACCCACAGAAACCATA | AGGTAAGGGCCATCTGAAAACT |
| Ass1 | ACACCTCCTGACTCCTCGT | GCTCACATCCTCAATGAACACCT |
| Aprt | CCCTCTTGAAAGACCCGGAC | CTGCGATGTAGTCGATCTTGC |
| β-actin | GTGCTATGTTGCTCTAGACTTCG | ATGCCACAGGATTCCATACC |
| Slc2a1 | CAGTTCGGCTATAACACTGGTG | GCCCCCGACAGAGAAGATG |
| LDHA | TGTCTCCAGCAAAGACTACTGT | GACTGTACTTGACAATGTTGGGA |
| LDHB | CATTGCGTCCGTTGCAGATG | GGAGGAACAAGCTCCCGTG |
| G6PC1 | CGACTCGCTATCTCCAAGTGA | GTTGAACCAGTCTCCGACCA |
| Fbp1 | CACCGCGATCAAAGCCATCT | AGGTAGCGTAGGACGACTTCA |
| Pck1 | CTGCATAACGGTCTGGACTTC | CAGCAACTGCCCGTACTCC |

**Supplemental Table 2 Antibodies and regents used in this paper**

| **REAGENT or RESOURCE** | **SOURCE** | **IDENTIFIER** |
| --- | --- | --- |
| **Antibodies** |  |  |
| Anti-L-Lactyl Lysine Rabbit mAb | PTM BIO Inc | Catalog # PTM  1401RM |
| PKM2-specific Polyclonal antibody | Proteintech | Cat No. 15822-1-AP |
| GLUT1 Polyclonal antibody | Proteintech | Cat No. 21829-1-AP |
| LDHA Polyclonal antibody | Proteintech | Cat No. 21799-1-AP |
| GLUT4 Monoclonal antibody | Proteintech | Cat No. 66846-1-Ig |
| Glut4 (1F8) Mouse mAb | Cell Signaling Technology | #2213 |
| LDHB Polyclonal antibody | Proteintech | Cat No. 14824-1-AP |
| NF-κB p65 Polyclonal antibody | Proteintech | Cat No. 10745-1-AP |
| Phospho-NF-κB p65 (Ser468) Recombinant antibody | Proteintech | Cat No. 82335-1-RR |
| JAK1 Monoclonal antibody | Proteintech | Cat No. 66466-1-Ig |
| JAK1 (phospho-Y1022) polyclonal antibody | Bioworld | BS4108 |
| STAT1 Polyclonal antibody | Proteintech | Cat No. 10144-2-AP |
| Phospho-STAT1 (Ser727) Polyclonal antibody | Proteintech | Cat No. 28977-1-AP |
| MCT1 Polyclonal antibody | Proteintech | Cat No. 20139-1-AP |
| MCT4 Polyclonal antibody | Proteintech | Cat No. 22787-1-AP |
| HIF-1 alpha Polyclonal antibody | Proteintech | Cat No. 20960-1-AP |
| mTOR Monoclonal antibody | Proteintech | Cat No. 66888-1-Ig |
| Phospho-mTOR (Ser2448) Monoclonal antibody | Proteintech | Cat No. 67778-1-Ig |
| β-Actin Rabbit mAb | ABclonal | AC026 |
| PerCP/Cyanine5.5 anti-mouse CD45 | BioLegend | 157207 |
| FITC anti-mouse CD3 | BioLegend | 100203 |
| APC anti-mouse CD4 | BioLegend | 116013 |
| Brilliant Violet 510™ anti-mouse CD8a | BioLegend | 100751 |
| Brilliant Violet 421™ anti-mouse CD19 | BioLegend | 115549 |
| PE anti-mouse/human CD45R/B220 | BioLegend | 103207 |
| Continued |  |  |
| **REAGENT or RESOURCE** | **SOURCE** | **IDENTIFIER** |
| Brilliant Violet 605™ anti-mouse/human CD11b | BioLegend | 101237 |
| PE anti-mouse F4/80 | BioLegend | 123109 |
| Zombie NIR™ Fixable Viability Kit | BioLegend | 423105 |
| APC anti-mouse CD11c | Biolegend | 117309 |
| **Reagents and Kits** |  |  |
| Sodium oxamate (Oxamate) | MCE | HY-W013032A |
| Fludarabine | MCE | HY-B0069 |
| Rapamycin | MCE | HY-10219 |
| Sodium L-lactate (Nala) | Sigma | 71718 |
| Phosphatase Inhibitor Cocktail | Analysis Quiz | AQ552 |
| Protease Inhibitor Cocktail (100×) | Analysis Quiz | AQ551 |
| RIPA Lysis Buffer | Beyotime | P013B |
| Deacetylase Inhibitor Cocktail | Beyotime | P1112 |
| DEPC water (DNase、RNase free) | Beyotime | R0021 |
| BeyoPure™ Ultrapure Water | Beyotime | ST873 |
| SDS-PAGE Separating Gel Buffer (4×) | CWBIO | CW0026S |
| SDS-PAGE Stacking Gel Buffer (4×) | CWBIO | CW0025S |
| TRIzol Reagent | Invitrogen | 15596026 |
| qRT-PCR primers, see Table S1. | This paper | N/A |
| FastKing RT kit (with gDNase) | TIANGEN | KR116 |
| Evo M-MLV RT Mix Kit with gDNA Clean for qPCR Ver.2 | Accurate Biology | AG11728 |
| SYBR Green Premix Pro Taq HS qPCR Kit （RoxPlus） | Accurate Biology | AG11718 |
| Pyruvate assay kit | Nanjing Jiancheng Bioengineering Institute | A081-1-1 |
| Pyruvate kinase assay kit | Nanjing Jiancheng Bioengineering Institute | A076-1-1 |
| Lactate dehydrogenase assay kit | Nanjing Jiancheng Bioengineering Institute | A020-2-2 |
| Glucose Assay Kit | Nanjing Jiancheng Bioengineering Institute | A154-1-1 |
| Lactic Acid assay kit | Nanjing Jiancheng Bioengineering Institute | A019-2-1 |

**
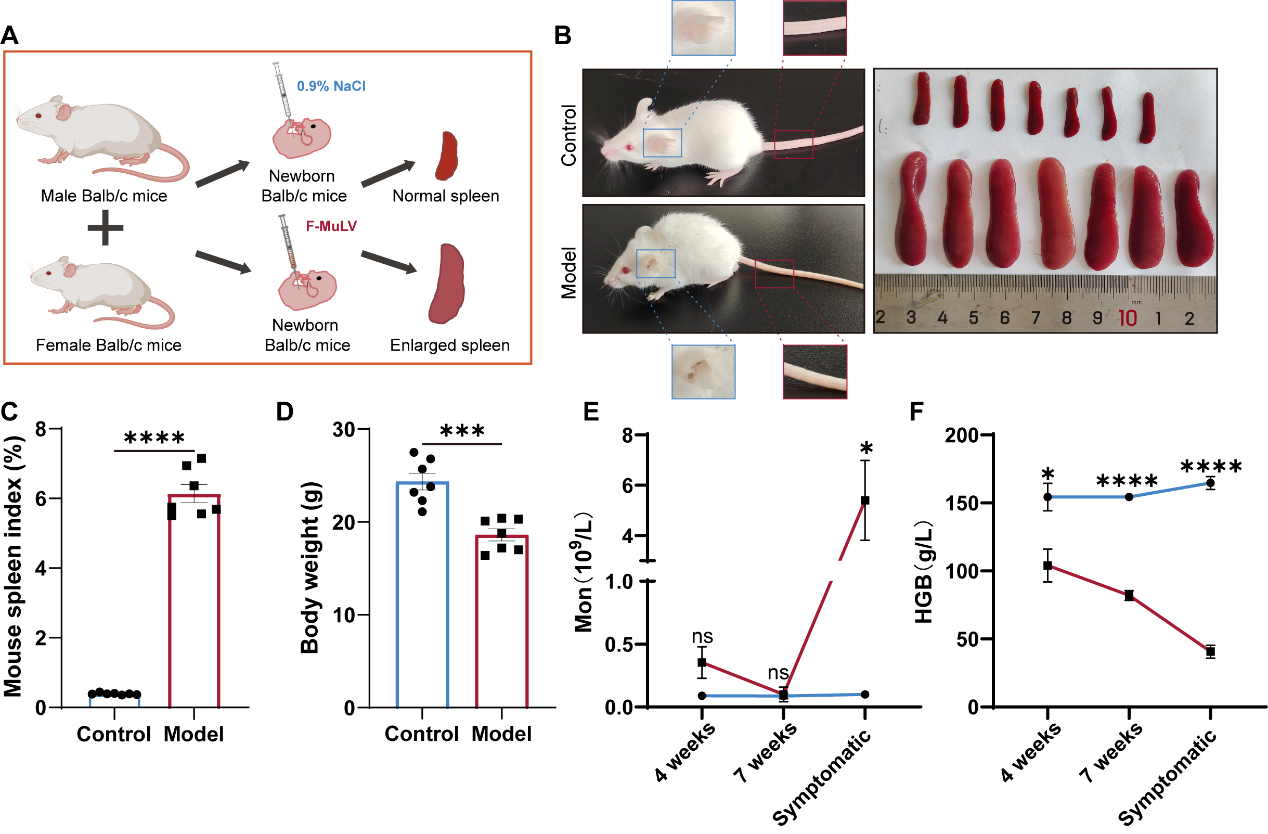
**

**Supplemental Figure 1.** **Construction of the AEL mouse model. A** Schematic of the F-MuLV-induced AEL model mice. **B** Representative images show a symptomatic mouse and its spleen; clinical signs included piloerection, a hunched posture, and pallor of the ear margins (blue box) and tail (red box). **C**-**D** Spleen index (**C**) and body weight (**D**) of AEL model mice; n = 7 mice per group. **E-F** Changes in peripheral blood monocyte count and hemoglobin level are shown. The data are presented as the means ± SEMs. Statistical significance is denoted as **p* < 0.05, ***p* < 0.01, ****p* < 0.001, and *****p* < 0.0001. Ns, nonsignificant.


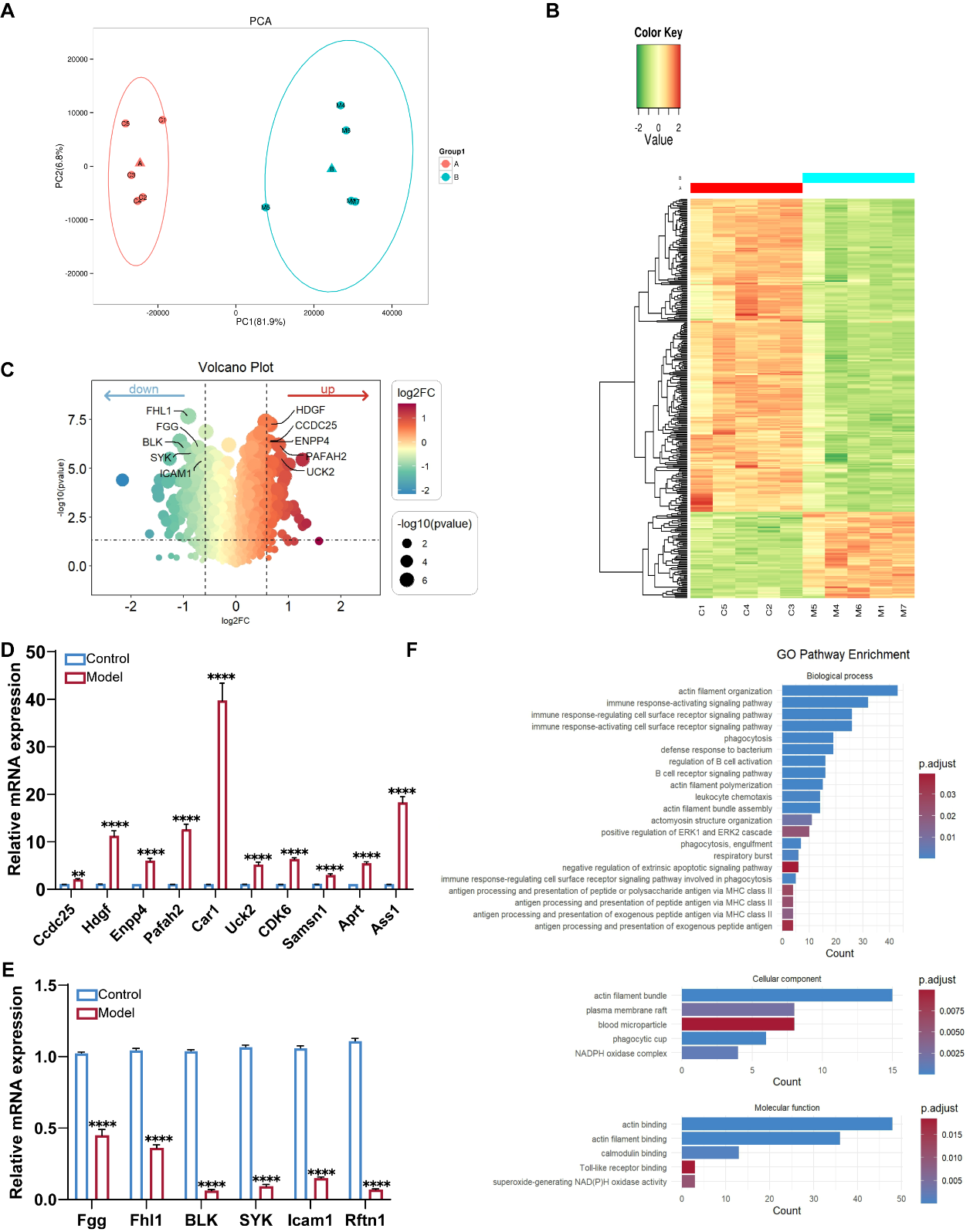


**Supplemental Figure 2.** **Validation of Proteomics Sequencing Data by qPCR. A** PCA of the normal and symptomatic AEL model mouse groups (8 weeks post virus injection). **B** Heatmap of differentially expressed proteins in the spleen tissues of normal and symptomatic AEL model mice. **C** Volcano plot visualizes these differentially expressed proteins. **D-E** qPCR analysis confirms the expression changes of genes encoding selected differentially expressed proteins in spleen tissues. **F** GO enrichment analysis was performed on the differentially expressed proteins. n = 5 mice per group in A -C and F. n = 3 mice per group in D and E. Data are presented as the means ± SEMs. **p* < 0.05, ***p* < 0.01, ****p* < 0.001, and *****p* < 0.0001.


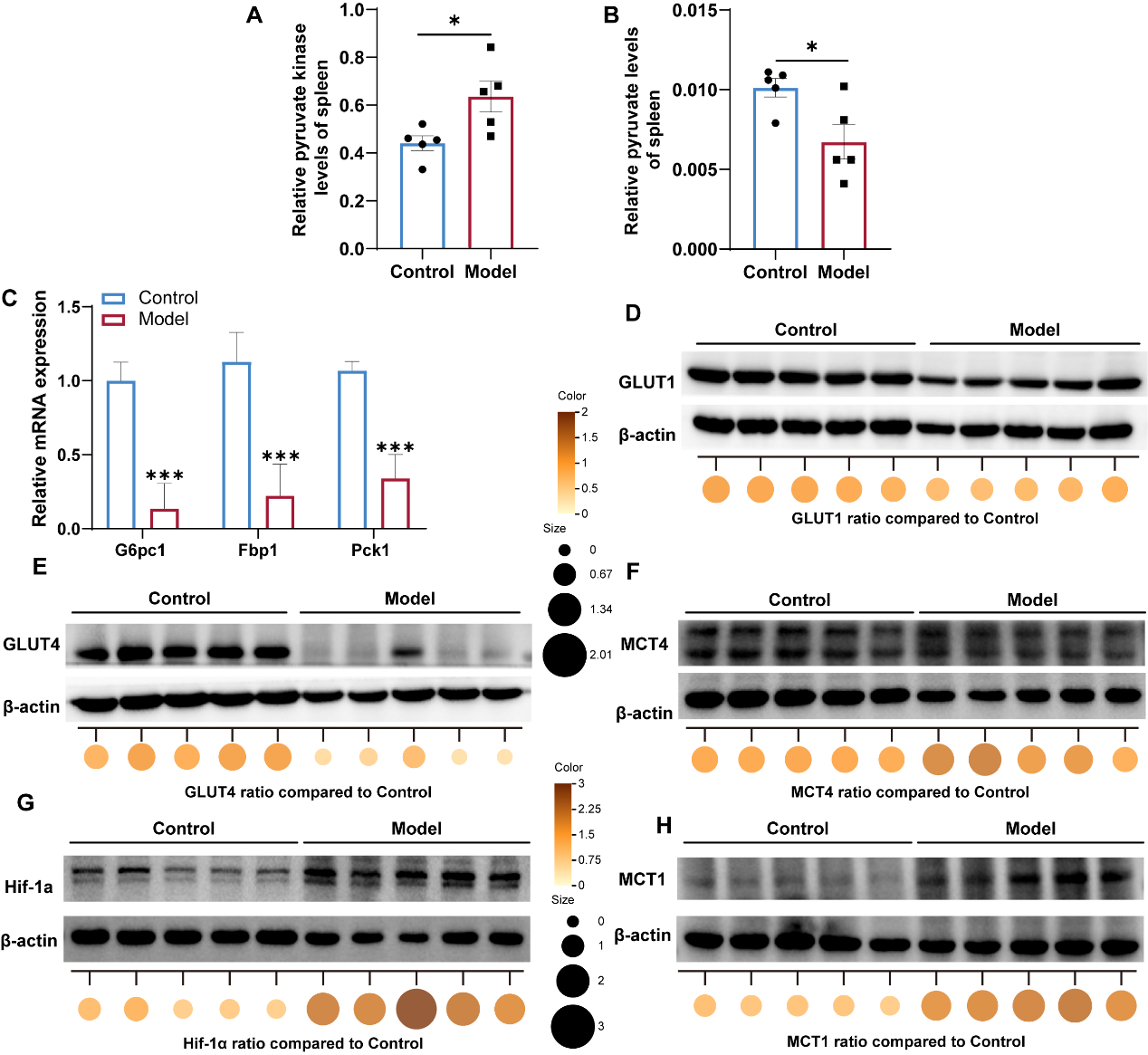


**Supplemental Figure 3.** **Alterations in glucose metabolism and lactate transport in the spleens of symptomatic AEL model mice. A** Splenic pyruvate kinase activity levels in AEL model mice; n = 5 mice per group. **B** Splenic pyruvate content in AEL model mice; n = 5 mice per group. **C** qPCR analysis of the mRNA expression levels of the key gluconeogenesis pathway genes *G6pc1*, *Fbp1*, and *Pck1*; n = 3 mice per group. **D-H** Western blotting and quantitative analysis of GLUT1, GLUT4, MCT4, Hif-1a, and MCT1 protein expression levels; n = 5 mice per group. The data are presented as the means ± SEMs. Statistical significance is denoted as **p* < 0.05, ***p* < 0.01, ****p* < 0.001, and *****p* < 0.0001.


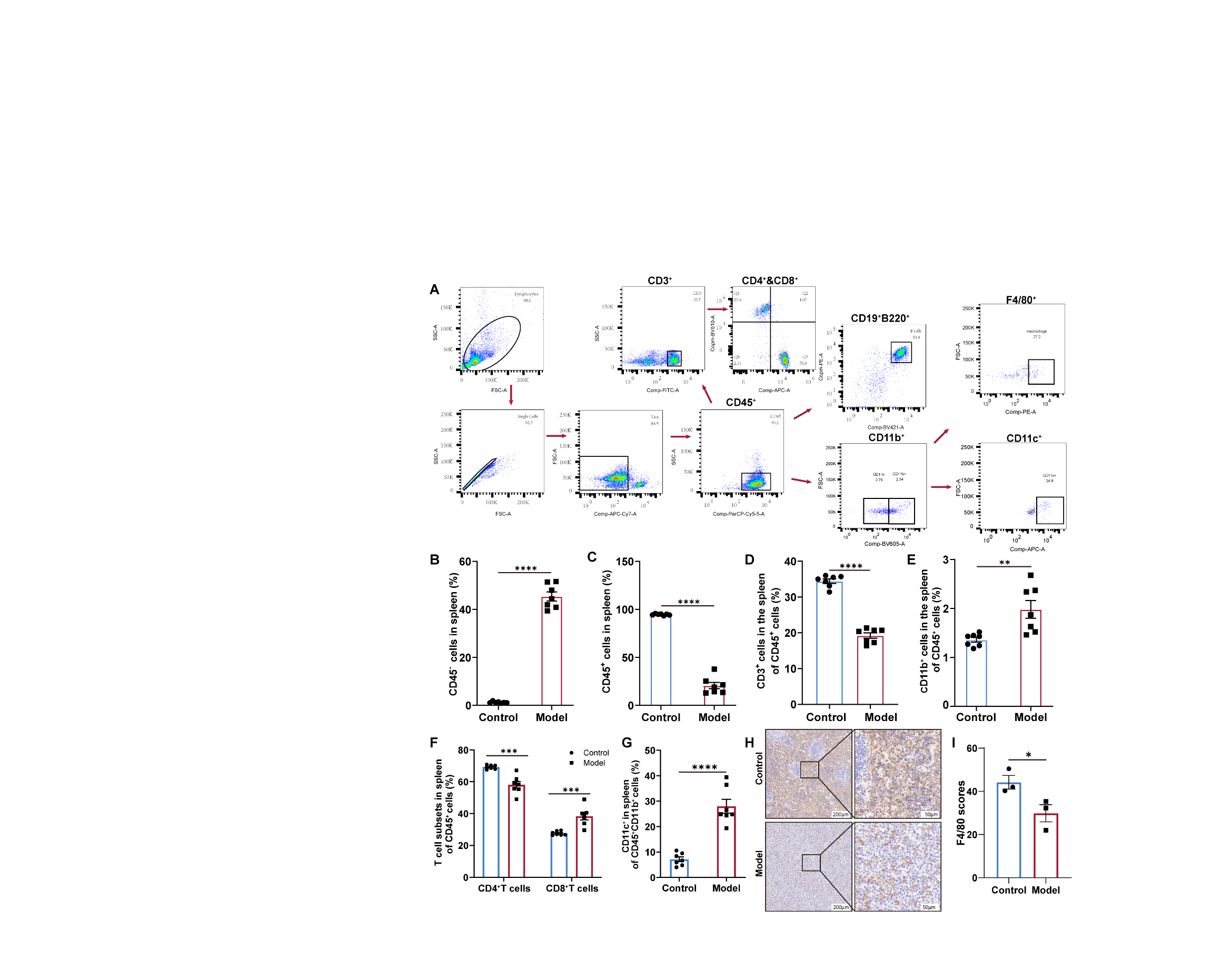


**Supplemental Figure 4.** **Immune cells in the spleens of AEL model mice exhibit abnormal expression.** **A** Gating strategy for flow cytometry analysis: live cells were initially gated, and total leukocytes were identified as CD45^+^ cells. From this population, T cells (CD3^+^), B cells (CD19^+^B220^+^), myeloid cells (CD11b^+^), dendritic cells (CD11b^+^CD11c^+^), and macrophages (CD11b^+^F4/80^+^) were subsequently distinguished. **B**-**G** Flow cytometry was used to analyze CD45^-^ cells (**B**), CD45^+^ cells (**C**), CD3^+^ T cells (**D**), CD11b^+^ myeloid cells (**E**), T cell subsets of CD4^+^ and CD8^+^ cells (**F**), and myeloid cells subset of CD11b^+^CD11c^+^ cells (**G**) isolated from the spleens of control and AEL symptomatic model mice; n = 7 mice per group. **H** Representative IHC images of F4/80, and statistical expression scores in **I** for the spleens of control and AEL model mice; n = 3 mice per group. Scale bars, 200 μm, 50 μm. These data are presented as the means ± SEMs. * *p* < 0.05, ** *p* < 0.01, *** *p* < 0.001, and **** *p* < 0.0001.


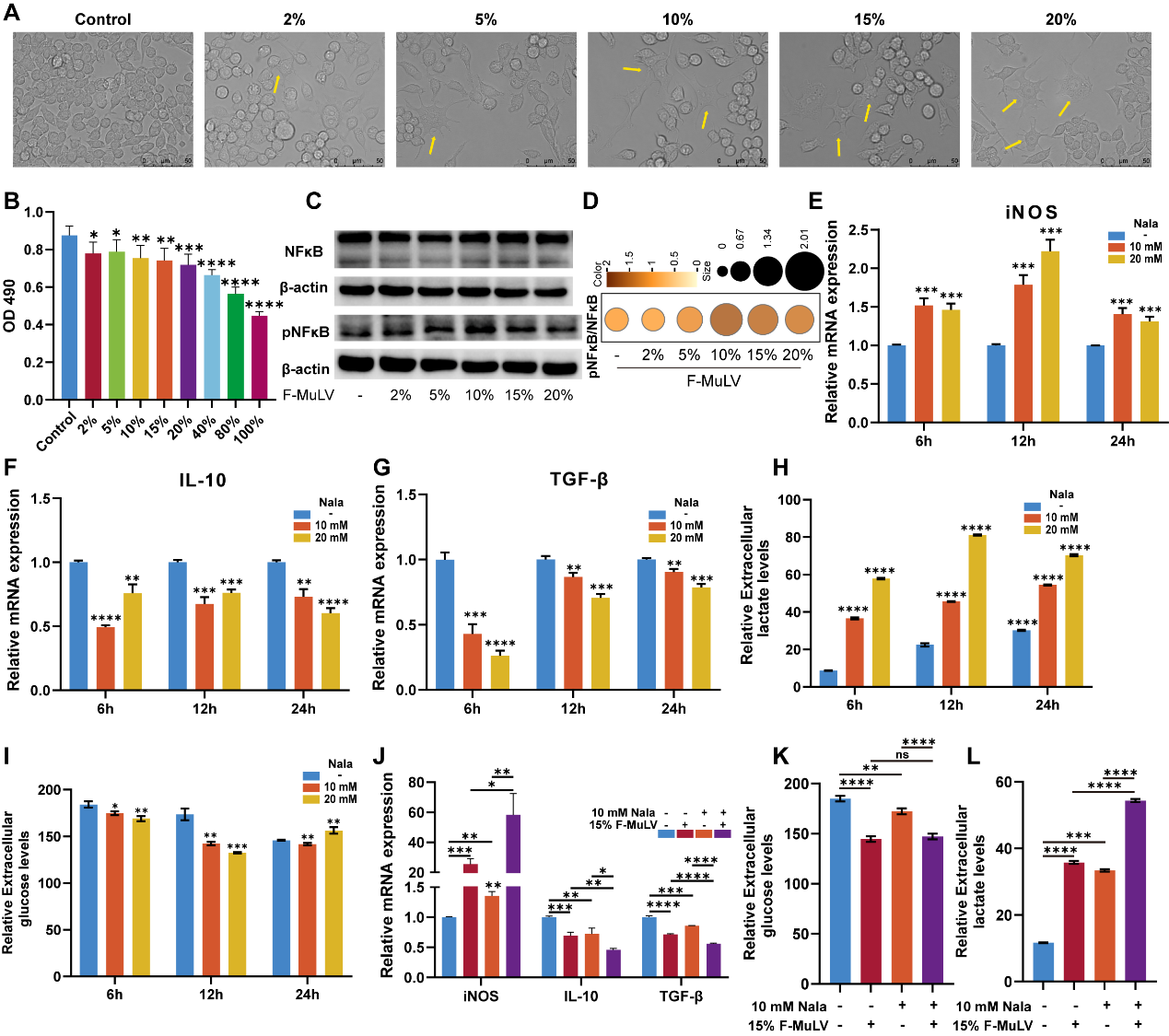


**Supplemental Figure 5. M1 polarization of macrophages in an *in vitro* model of AEL. A** Morphology of RAW264.7 macrophages following 24-hour treatment with the indicated concentrations of F-MLV; scale bars, 50 μm. **B** Viability of RAW264.7 macrophages after 24-hour exposure to different concentrations of F-MuLV. **C-D** Western blotting and quantitative analysis of the protein expression levels of NFκB and pNFκB. **E**-**G** qPCR analysis of the mRNA expression levels of *iNOS*, *IL-10*, and *TGF-β* in RAW264.7 macrophages treated with Nala at the specified concentrations for 6, 12, and 24 hours. **H**-**I** Lactate and glucose levels measured in the supernatants under the treatment conditions described for panels **E**-**G**. **J**-**L** qPCR analysis of the mRNA expression levels of *iNOS*, *IL-10*, and *TGF-β* in RAW264.7 macrophages treated with 15% F-MuLV with or without 10 mM Nala for 12 hours (**J**). The corresponding lactate (**K**) and glucose (**L**) concentrations in the culture supernatants are presented. These data are presented as the means ± SDs. * *p* < 0.05, **** *p* < 0.01, *** *p* < 0.001, and **** *p* < 0.0001; ns, nonsignificant.


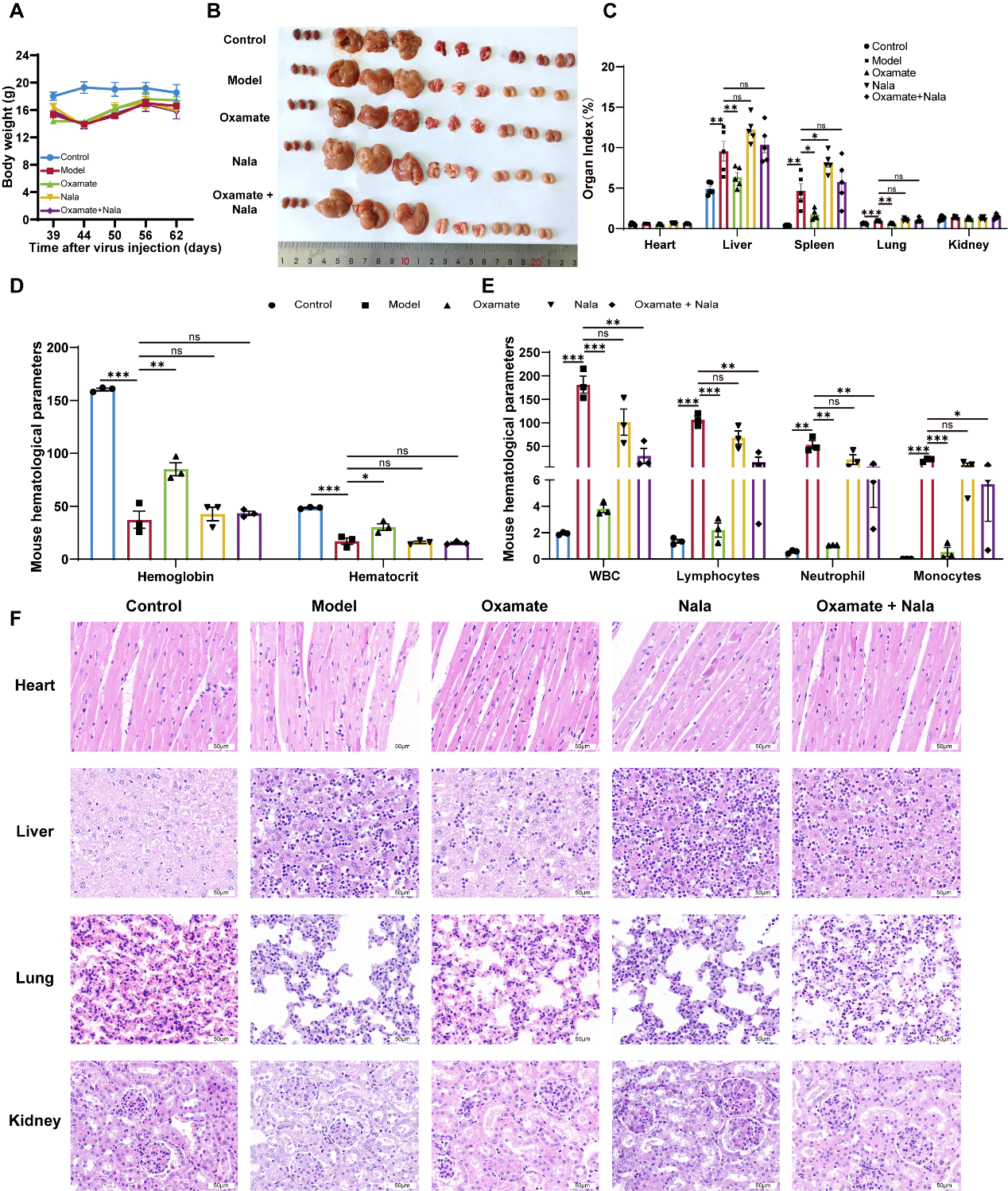


**Supplemental Figure 6.** ***In vivo* inhibition of lactate production effectively alleviated organ abnormalities in AEL model mice. A** Body weight changes of the mice in each group during the administration period; n = 5 mice per group. **B** Macroscopic appearance of the heart, liver, lungs, and kidneys in the control group, model group, Oxamate group, Nala group, and Oxamate + Nala group. **C** Organ indices of the heart, liver, spleen, lung, and kidney in the mice from each group; n = 5 mice per group. **D-E** Routine blood examination of each group; n = 3 mice per group. **F** Representative H&E-stained images of heart, liver, lung, and kidney tissues from mice in each group; n = 3 mice per group. Scale bars, 50 μm. These data are presented as the means ± SEMs. * *p* < 0.05, **** *p* < 0.01, *** *p* < 0.001, and **** *p* < 0.0001; ns, nonsignificant.
