# Supplementary figures and images for: Lactate-mediated macrophage polarization promotes splenomegaly in acute erythroleukemia
Source: Cell Death Dis. 2026 Mar 25;17(1):373. doi: 10.1038/s41419-026-08612-5 (PMC13039512; doi:10.1038/s41419-026-08612-5)

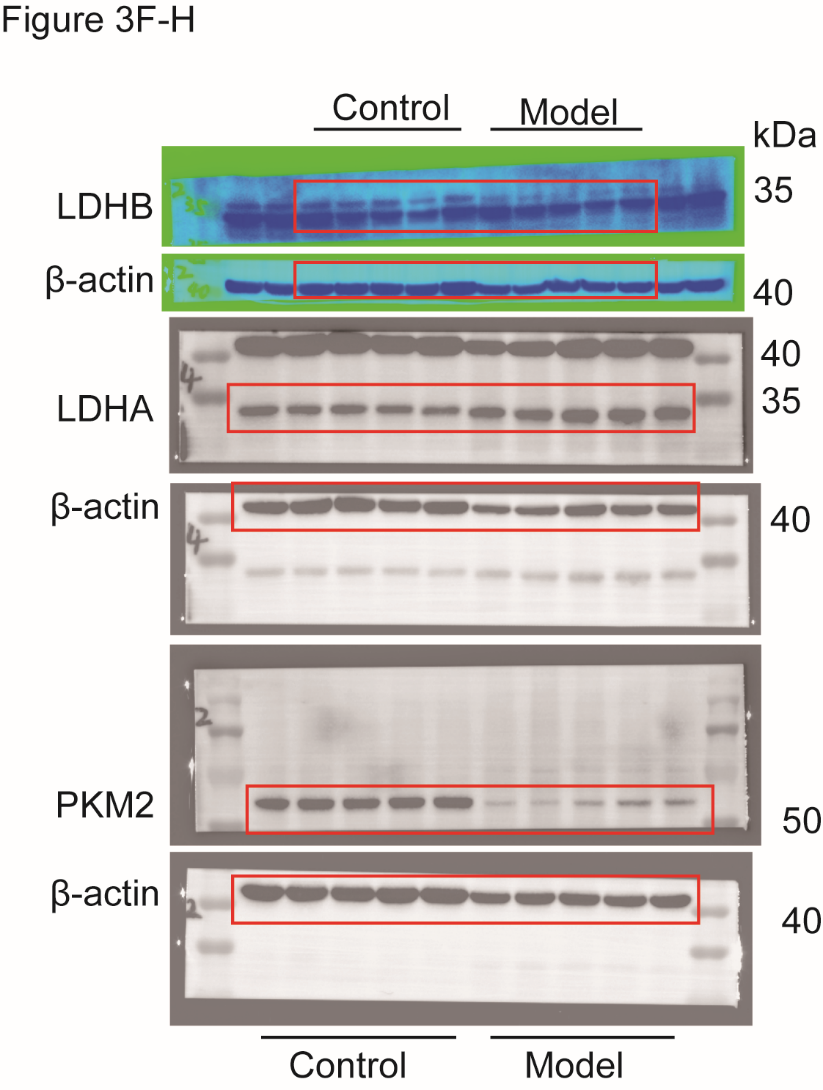


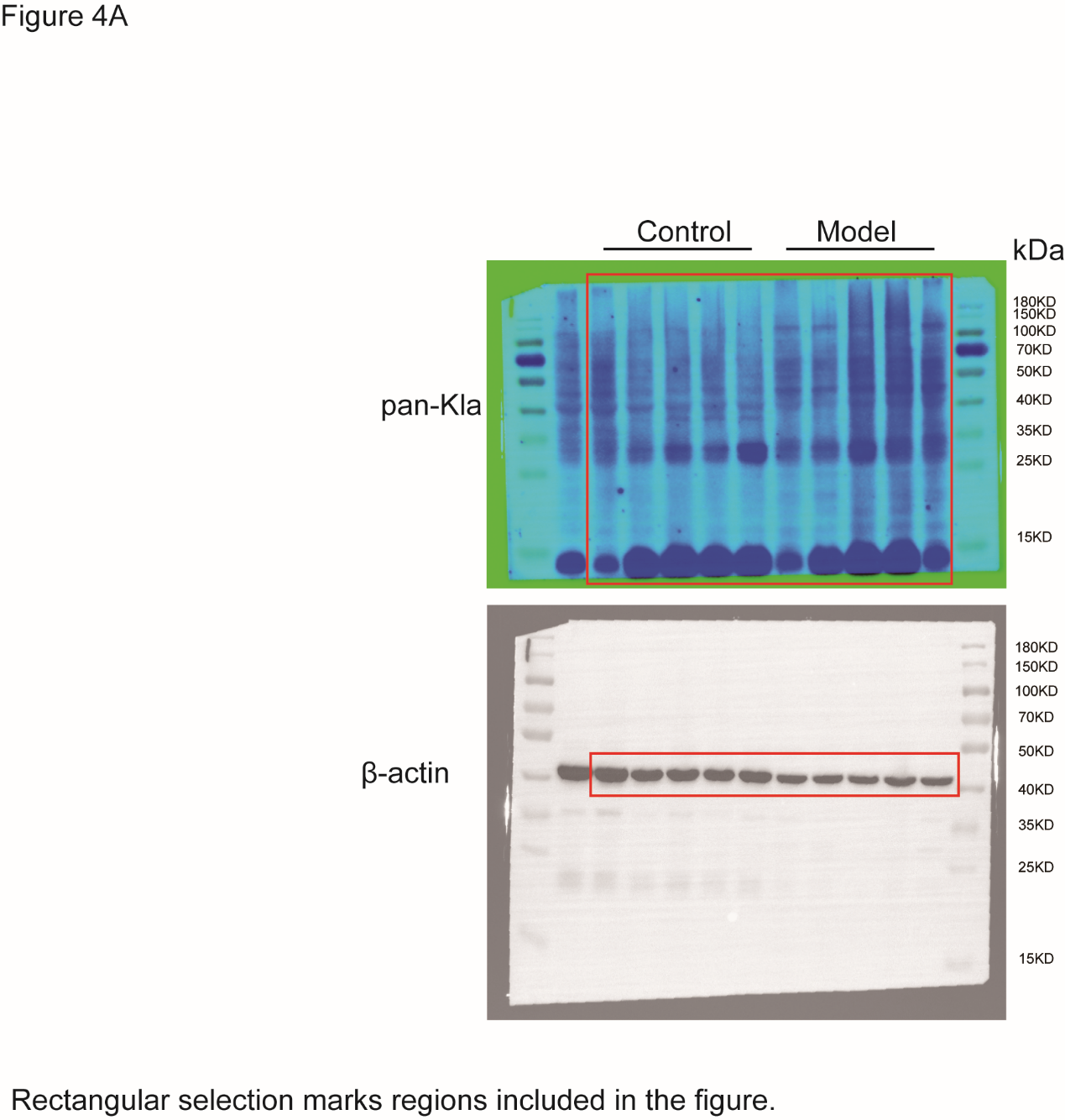


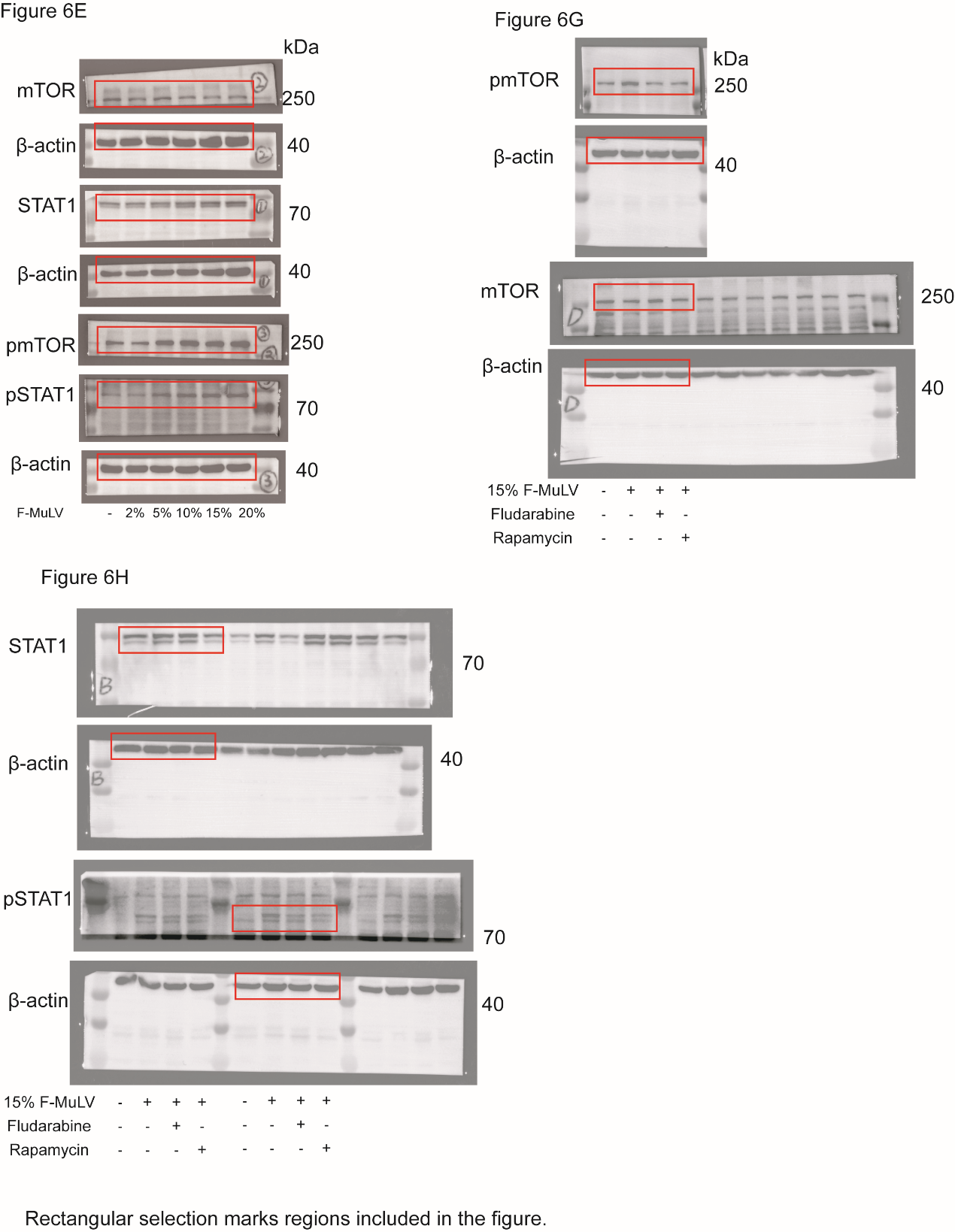


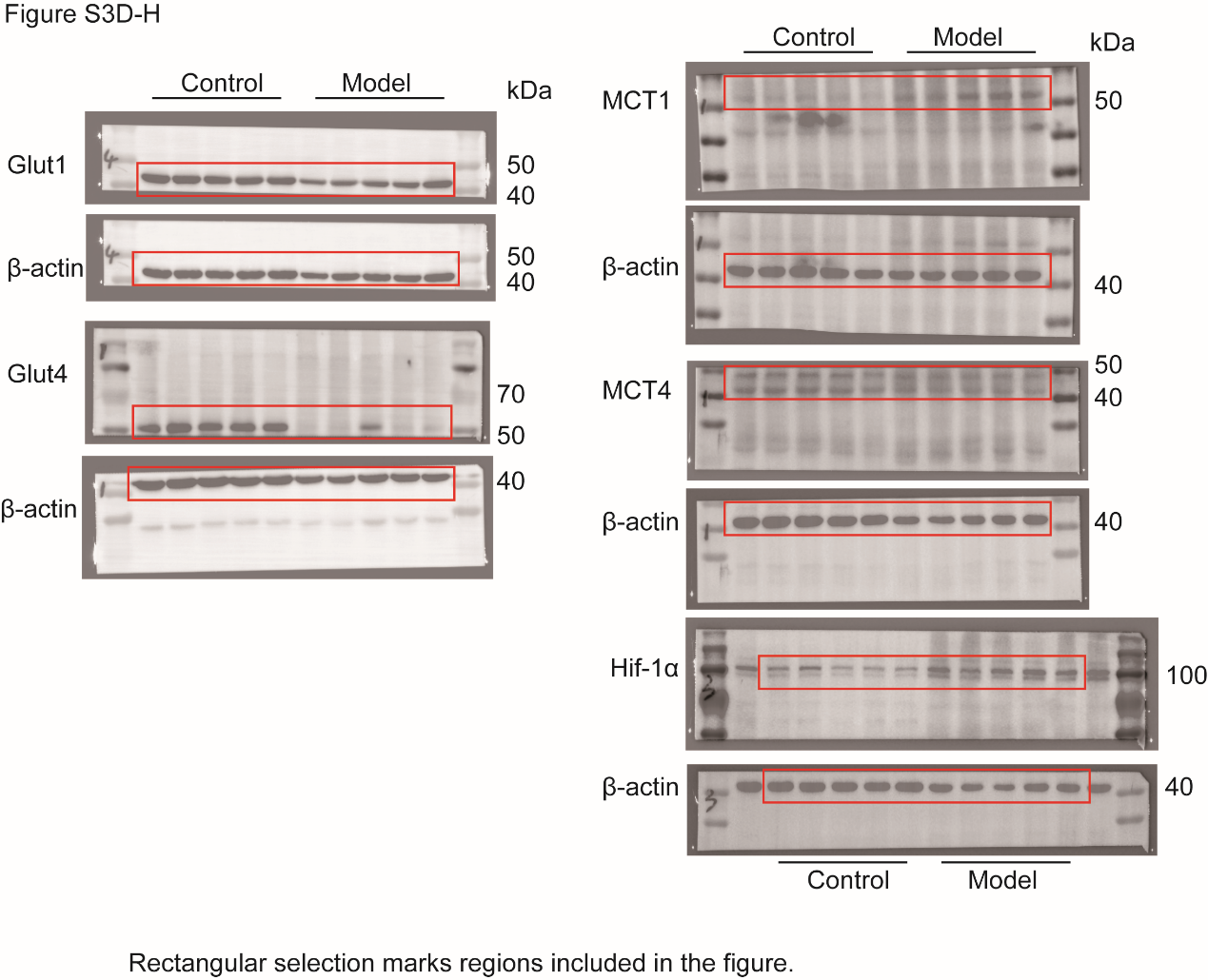


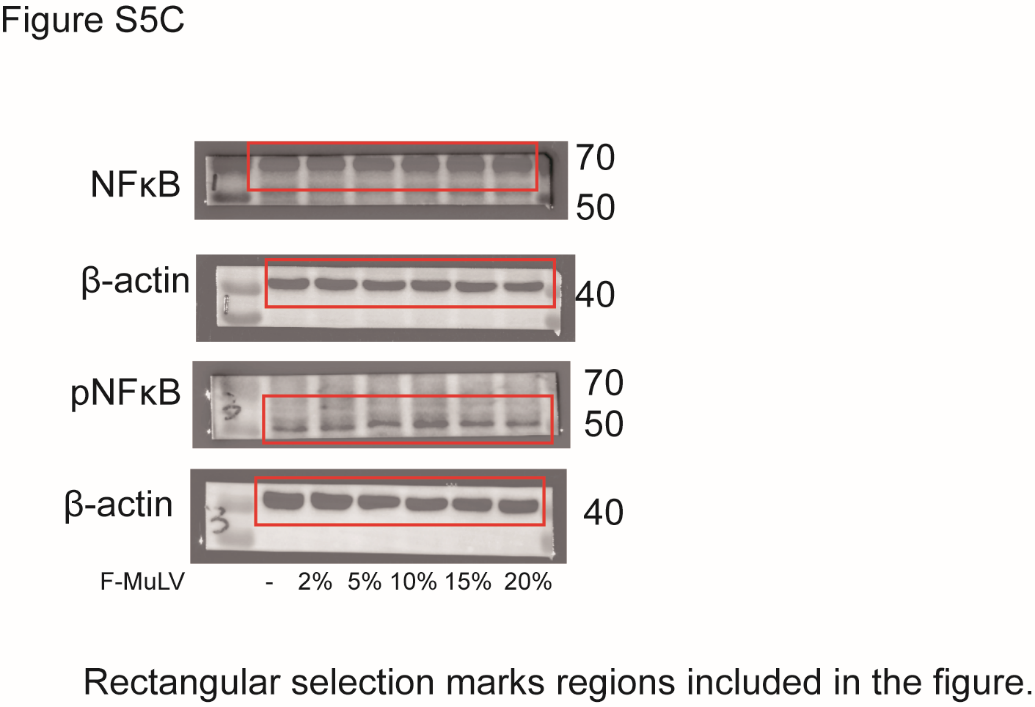

Supplement: Supplementary file 2 — Original Western blots [file 41419_2026_8612_MOESM2_ESM.docx]
